# How Many Participants Do We Have to Include in Properly Powered Experiments? A Tutorial of Power Analysis with Reference Tables

**DOI:** 10.5334/joc.72

**Published:** 2019-07-19

**Authors:** Marc Brysbaert

**Affiliations:** 1 Department of Experimental Psychology, Ghent University, BE

**Keywords:** Statistical analysis, Mathematical modeling, Working memory

## Abstract

Given that an effect size of d = .4 is a good first estimate of the smallest effect size of interest in psychological research, we already need over 50 participants for a simple comparison of two within-participants conditions if we want to run a study with 80% power. This is more than current practice. In addition, as soon as a between-groups variable or an interaction is involved, numbers of 100, 200, and even more participants are needed. As long as we do not accept these facts, we will keep on running underpowered studies with unclear results. Addressing the issue requires a change in the way research is evaluated by supervisors, examiners, reviewers, and editors. The present paper describes reference numbers needed for the designs most often used by psychologists, including single-variable between-groups and repeated-measures designs with two and three levels, two-factor designs involving two repeated-measures variables or one between-groups variable and one repeated-measures variable (split-plot design). The numbers are given for the traditional, frequentist analysis with p < .05 and Bayesian analysis with BF > 10. These numbers provide researchers with a standard to determine (and justify) the sample size of an upcoming study. The article also describes how researchers can improve the power of their study by including multiple observations per condition per participant.

*Statistical packages tend to be used as a kind of oracle …. In order to elicit a response from the oracle, one has to click one’s way through cascades of menus. After a magic button press, voluminous output tends to be produced that hides the [critical information] …, among lots of other numbers that are completely meaningless to the user, as befits a true oracle.* (*Baayen, 2008, viii*)

Baayen’s (2008) observation about psychologists’ use of statistical software packages is probably nowhere more relevant than for the calculation of a study’s power and the minimum number of participants required for a properly powered study.^1^ The power of a study roughly refers to the chances of finding an effect in a study given that it exists in reality (at the population level). Cohen (1992, p. 156) defined the statistical power of a significance test as the long-term probability of rejecting the null hypothesis, given the effect size in the population, the chosen significance level, and the number of participants tested.

Fraley and Vazire (2014) summarized the problems associated with underpowered studies. First, low power studies are less likely to find a true effect (i.e., there is no statistical significant effect in the study, even though the effect exists at the population level). Second, true effects that are detected tend to have inflated effect sizes (i.e., a true effect is only significant in an underpowered study when the effect obtained in the study is larger than the effect at the population level). At the same time, when a statistically significant effect is found, chances that it is a false positive are higher in underpowered studies than in well-powered studies (i.e., the effect found in the study is a fluke that does not exist in reality). As a result, findings from low-powered studies are less replicable.

Even seasoned researchers struggle to understand the minutiae of power analysis for the designs they are using. The same is true for editors and reviewers trying to judge the adequacy of a submitted manuscript or grant proposal. Many authors currently refer to the outcome of some software package, claiming that the power of their design is .80 at least, whereas some simple calculations make such estimate highly unlikely. Two examples of manuscripts and grant applications I was recently asked to review, are:

- *An a priori power analysis for a repeated-measures analysis of variance that examined main effects and interactions with two groups and five repeated-measures showed that 15 participants in each group would provide greater than 80% power (α = .05) to detect a medium effect (η^2^_p_ = .05) in our dependent measures of interest*.
- *As the groups are not large (N = 25) we used ANCOVA because this is more powerful*.

Both statements are tricky, because they contain a kernel of truth, but at the same time are hopelessly overoptimistic, as we will see later.

In the present text, I discuss guidelines that can be used to evaluate studies and set up new good studies. Indeed, one of the big frustrations for people searching the literature on statistical power is that very few publications give explicit target numbers. They all point to the importance of the (unknown) effect size and then leave it to the readers to calculate the required numbers themselves, bringing the readers back to square one. So, I will stick out my neck and give specific target numbers for various designs, based on some reasonable and explicit assumptions.^2^ These target numbers (summarized in Tables 7, 8, 9) provide an explicit point of reference against which to determine and justify the sample size of an upcoming study. Before turning to the numbers I give some background information.

## Psychological researchers have a pathological fear of overpowered studies

One of the recurrent questions psychology researchers ask is: “What is the *minimum* number of participants I must test”? They ask this not because they want to play safe and run more participants than minimally required, but because they want to have an idea of the *maximum* number of participants they should run. Indeed, very few researchers intently run studies that include more than the minimum required.^3^ The following five reasons seem to be responsible for this bias.

The first reason is the prevailing culture and education of psychology researchers. Running more participants than strictly needed is considered waste. To some extent this is true (though see below), but the history of psychological research has shown that in reality it leads to an excess of underpowered studies. Such excess has been the case for decades and keeps on being true for many studies run today (Cohen, 1962; Dumas-Mallet, Button, Boraud, Gonon, & Munafò, 2017; Fraley & Vazire, 2014; Maxwell, 2004; Szucs & Ioannidis, 2017a; Vankov, Bowers, & Munafo, 2014), despite the fact that many articles on power calculation and required sample sizes have been published (e.g., Cohen, 1988, 1992; Fraley & Vazire, 2014; Lachin, 1981; Maxwell, 2004; Murphy, Myors, & Wolach, 2014; Tomczak, Tomczak, Kleka, & Lew, 2014; Wilkinson, 1999). To be clear, running a few more participants than strictly needed on the basis of power analysis involves a minor financial cost, whereas running fewer participants entails an increased risk of drawing incorrect conclusions.

The second reason for underpowered studies is that up to recently we tended to underestimate the number of data needed. For simple designs the numbers have been known for a long time (although often not taken into account!), but we have wrongly assumed that the situation is less strict for complicated designs with crossed variables. In some situations it is true that one can save some efficiency by crossing variables, but in general researchers have hugely underestimated the number of observations needed for interactions (see below).

The third reason is that properly powered studies rarely get a quality label and that underpowered studies have hardly been at a disadvantage to get published. Indeed, up to recently, power issues had a very low weight in the evaluation of results. As long as an effect was significant, it was assumed that the study had enough power (even had too much power if p < .001) and reviewers who nevertheless raised power issues were usually discarded as overzealous. Power was an issue only when effects were not significant. Similarly, a study did not have more chances of being accepted for publication because it compared two groups of 100 participants, rather than two groups of 20 participants.^4^ As a result, there has been little incentive to run properly powered studies (Edwards & Roy, 2017; Higginson, & Munafò, 2016; Munafò, et al., 2017; Smaldino & McElreath, 2016).

Fourth, researchers seem to overestimate the effect sizes they are examining and have overly optimistic intuitions about the numbers needed for properly powered experiments (Bakker, Hartgerink, Wicherts, & van der Maas, 2016).^5^

Finally, researchers seem to be happy as long as they obtain significance for some effects in rather complicated designs, even though these effects were not predicted and are unlikely to be replicated. This is because designs with several tests have a high chance of yielding at least one statistically ‘significant’ outcome. Maxwell (2004, Table 7) illustrated this with a 2 × 2 between-groups factorial design analyzed with ANOVA. The effects of both variables and the interaction were medium (i.e., d = .5). Even with a massively underpowered study involving groups of only 10 participants, simulations indicated that at least one of the effects was significant in 71% of the simulations! Virtually none of these simulations (4%) indicated that all effects were significant, as should have been found in a properly powered experiment. Instead, the simulations randomly pointed to one of the effects being significant (A, B, or A × B: 15% each), or to two of the three effects being significant (8% each). As Maxwell (2004) argued, a sequence of such studies gives each researcher the illusion of having discovered something, but leads to a messy literature when authors try to decide which variables influence behavior and which do not.

## Properly powered studies will often look needlessly overpowered

For many psychological researchers, a properly powered study is a study in which an expected effect is significant at p < .05. Effects with a significance level of p < .001 feel ‘needlessly overpowered’. This is the result of two misconceptions.

The first misunderstanding is that an effect significant at p < .05 has 95% chance of being replicated if the study is run again in exactly the same way. This is not true. The probability of successful replication for such a study is only 50% (Vankov et al., 2014).

The second misunderstanding is that power only has implications for p-values around .05. Power informs us about the chances of the results turning out against us to such an extent that we fail to find significance, even though the effect exists at the population level. However, power has an effect on the complete range of p-values. Let us illustrate this with two hypothetical studies each having one repeated-measures factor with two levels (i.e., one of the simplest designs possible). In the first study, there is no effect at the population level (d = 0). In the second study, there is an effect of d = .4 at the population level.

There are three scenarios for each study: (i) with 10 participants, (ii) with 30 participants, and (iii) with 100 participants. From a basic stats course, we know that the condition means on average will coincide with the population values, but that there is likely to be some deviation from study to study because of sampling error. The smaller the number of participants, the larger the deviations (you may even remember that the standard error of the mean is the standard deviation divided by the square root of the number of participants tested).

**Figure 1:**
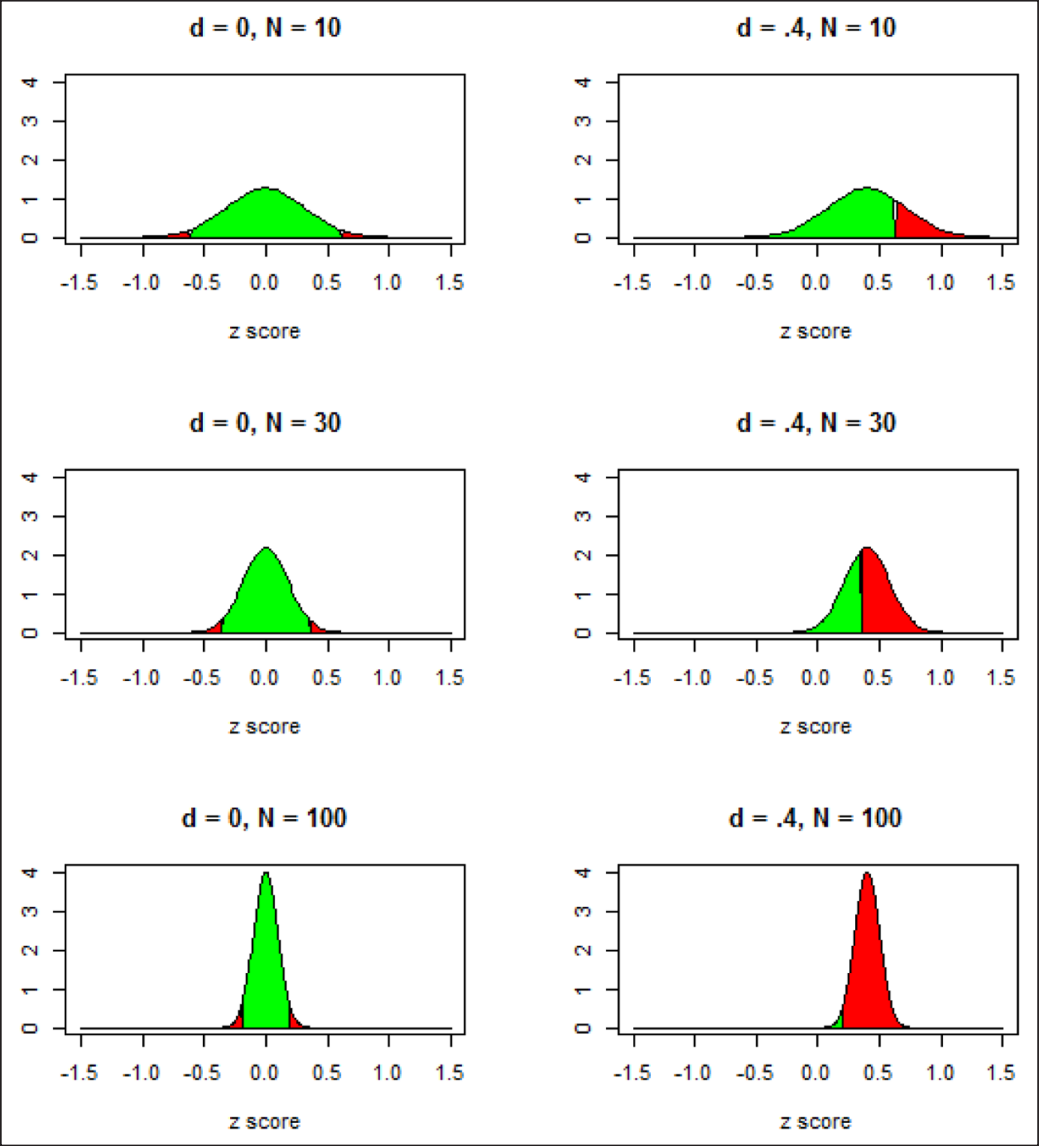
What happens to the significance of an effect when a study becomes more powerful? Red areas are p < .05, two-tailed t-test; green area is not significant.

Figure 1 illustrates what the different sample sizes entail for the effect sizes (the difference between the means of the two conditions) that can be expected. For the small sample sizes (N = 10) there is quite some scatter in the effects that will be obtained from study to study; for the large sample sizes (N = 100) the scatter is much less. Each panel of Figure 1 also indicates the chances that the researcher will obtain a significant finding (p < .05, two-tailed), both when there is no effect in the population and when there is an effect of d = .4.

From Figure 1 we can derive the distribution of p-values a researcher would observe if they repeated the study many times. These are listed in Table 1. In this table we see that even for an underpowered study with N = 30 (49% power) we expect to find p < .01 in 35% of the studies (of which 14% will be p < .001). For an overpowered study of N = 100 (98% power), the vast majority of p-values will be smaller than .001. However, even here we see that the researcher has 7% chance of observing a p-value > .01 (of which 2% will be > .05). For a reasonably powered study (N = 52, 80% power), chances of finding p < .01 are 60%, of which 32% will be p < .001. These numbers of “overly significant effects” are to be expected in a properly powered study, but tend to be interpreted by researchers as a license to decrease the number of participants. It is good to keep in mind that a series of studies all returning p-values just below .05 is not a sign of properly powered studies, but a warning that researchers have omitted studies with nonsignificant findings or massaged their data (Francis, 2012; John, Loewenstein, & Prelec, 2012). Properly powered studies often return p < .001, just like in 20% of the cases they return non-significant results. This is a consequence of noise in the data, which causes variation in the outcome of experiments run repeatedly.

**Table 1:** The outcome in terms of p-values a researcher can expect as a function of the effect size at the population level (no effect, effect of d = .4) and the number of participants tested in a two-tailed test. The outcome remains the same for the sample sizes when there is no effect at the population level, but it shifts towards smaller p-values in line with the hypothesis when there is an effect at the population level. For N = 10, the statistical test will be significant at p < .05 in 15 + 7 + 2 = 24% of the studies (so, this study has a power of 24%). For N = 30, the test will be significant in 24 + 21 + 14 = 49% of the studies. For N = 100, the test will be significant for 6 + 16 + 76 = 98% of the studies, of which the majority with have significance at p < .001. At the same time, even for this overpowered study researchers have 7% chance of finding a p-value hovering around .05.

	N = 10		N = 30		N = 100	

	d = 0	d = .4	d = 0	d = .4	d = 0	d = .4

p < .001 against hypothesis	0.0005	≈0%	0.0005	≈0%	0.0005	≈0%
.001 ≤ p < .01 against hypothesis	0.0045	≈0%	0.0045	≈0%	0.0045	≈0%
.01 ≤ p < .05 against hypothesis	0.0200	0.0006	0.0200	≈0%	0.0200	≈0%
.05 ≤ p < .10 against hypothesis	0.0250	0.0012	0.0250	0.0142	0.0250	≈0%
p ≥ .10 against hypothesis	0.4500	0.1011	0.4500	0.2783	0.4500	≈0%
p ≥ .10 in line with hypothesis	0.4500	0.5451	0.4500	0.1162	0.4500	0.0092
.05 ≤ p < .10 in line with hypothesis	0.0250	0.1085	0.0250	0.2412	0.0250	0.0114
.01 ≤ p < .05 in line with hypothesis	0.0200	0.1486	0.0200	0.2412	0.0200	0.0565
.001 ≤ p < .01 in line with hypothesis	0.0045	0.0735	0.0045	0.2144	0.0045	0.1618
p < .001 in line with hypothesis	0.0005	0.0214	0.0005	0.1357	0.0005	0.7610

## Published effect sizes are likely to be overestimates

Statistics are particularly trustworthy when you have lots of data or lots of studies by independent researchers. However, most of the time we have only a few studies (or even only one) rather than the thousands implied in Figure 1. In such a situation, we try to deduce the nature of the population from the few data points (effects) we have. This is complicated by two issues.

The first issue is that not all studies are available in the literature. Every day thousands of studies are run by undergraduates, master students, PhD students, postdocs, academic staff, retired academics, and the occasional independent researcher. Not all of these studies can be published (can they?). Some of them were badly designed; others did not yield results of interest. The latter, however, is a problem, because it introduces publication bias. Given that null results are generally seen as uninteresting, there is a bias to publish significant results (a tendency that is present in those who ran the study, as well as in editors and reviewers deciding whether the study is interesting enough to be published).

In the extreme case, publication bias can lead to a lot of ‘scientific’ discussion without insight. Suppose a world in which all hypotheses examined by researchers are wrong (there are no effects at the population level) but only the experiments with significant results get published. (Remember that Maxwell, 2004, found at least one ‘significant’ effect in 71% of the studies with a 2 × 2 design, even though each study was heavily underpowered; also see Bishop, 2013, related to this problem). The literature then would entirely consist of papers with exciting, ‘significant’ findings (often with p < .001). However, the findings would not be replicable and would contradict each other, certainly when the researchers use two-tailed significance tests, because half of the published effects would go in one direction, and the other half go in the other direction, independent of how powerful the tests are (left column of Figure 1).

**Figure 2:**
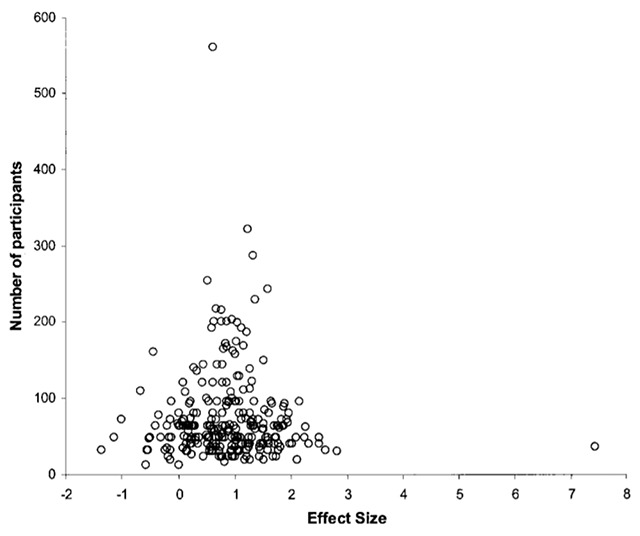
Tens of studies on the difference in vocabulary size between old and young adults. Positive effect sizes indicate that old adults know more words than young adults. Each circle represents a study. Circles at the bottom come from studies with few participants (about 20), studies at the top come from large studies (300 participants or more). Source: Verhaeghen (2003).

Fortunately, the situation is not as bad as in the above thought experiment (at least we hope so; but see Johnson, Payne, Wang, Asher, & Mandal, 2017, for a sobering analysis). Psychologists have stable theories that lead to valid predictions, and findings are replicated (sometimes). However, the effect of publication bias is there and has an effect that can be demonstrated. One of the consequences of publication bias is that the standardized effect size of published studies is too large, particularly when the effect is repeatedly studied with small samples (Maxwell, 2004). If a certain research tradition only studies a repeated-measures factor with samples of N = 20, we can expect that all published effect sizes are d > .47. Otherwise, the test is not significant (and thus not published). Indeed, Kühberger, Fritz, and Scherndl (2014, Figure 4) reported that the average effect size of published studies was considerably higher for studies with less than 50 participants than for studies with more than 500 participants. Publication biases can also be seen in meta-analyses, when funnel plots are used (see Figure 2 below).

The second reason why published effect sizes are likely to be overestimates, is that researchers have lots of incentives to decrease p-values of almost significant results and none to increase p-values of significant findings (John et al., 2012). Because p-values are inversely related to effect sizes, these practices will lead to higher published effect sizes than warranted if the noise had not been tinkered with. The classic reference to this practice, called p-hacking, is Simmons, Nelson, & Simonsohn (2011). They showed that creative researchers can turn an alpha level of 5% into an alpha level of more than 30% (i.e., 30% chances of accepting an effect that is not there at the population level).

The end result of publication bias and p-hacking is that published findings usually are not a good source to estimate the size of the effect you are going to examine (remember that power programs critically rely on this information).

## Pilot studies are next to worthless to estimate effect sizes

Another potential source for estimating the effect size comes from data of a small-scale study you already ran. Indeed, grant submissions have more chances of being awarded if they include pilot testing. Pilot testing is good for showing the feasibility of a technique and for trying out the procedure but, unfortunately, do not provide reliable information when it comes to estimating effect sizes (also see Albers & Lakens, 2018; Kraemer, Mintz, Noda, Tinklenberg, & Yesavage, 2006). Even worse, pilot testing is likely to put you on a false trail if a significant effect in pilot testing is the only reason to embark on a project.

Pilot testing does not give valid estimates of effect sizes for the simple reason that they are too small. Everything we discussed related Figure 1 is also true for pilot testing. To illustrate the issue, let’s think of the following question. Do old adults know more words than young adults? (Before continuing, you may want to think what your answer to that question is).

The nice aspect about the question is that there are many data around. In many studies, young adults are compared to old adults, and quite often a vocabulary test is included to get an estimate of the crystallized intelligence of the participants. So, all we have to do is to search for studies including vocabulary tests and write down (1) the sample sizes tested, and (2) the effect size reported. Verhaeghen (2003) did exactly this analysis. Figure 2 shows the outcome.

Two aspects are noteworthy in Figure 2. First, it looks like old adults know more words than young adults. Second, and more important for the present discussion, when one runs a small-scale study (N ≈ 20), one can expect standardized effect sizes ranging from d = –1.5 (indicating a strong advantage for young adults) to d = 2.5 (an even stronger advantage for old adults). This illustrates the problem you are confronted with when you run a single, small-scale pilot study. You have no idea where your single data point falls relative to the entire picture. So, finding d = 1 is as uninformative as finding d = –1 or d = 0. All you can do (and should do) is to calculate the confidence interval around the effect size (Fritz, Morris, & Richler, 2012). This will inform you that with small samples the confidence intervals around obtained effect sizes cover almost all effect sizes going from big effect sizes in favor of the hypothesis to big effect sizes against the hypothesis. Hopefully this will save you from overinterpreting effect sizes obtained in pilot studies.

Another nice aspect of Figure 2 is that the question was theory neutral. The data had not been collected to test one or the other theory; they were just collected for descriptive purposes. As a result, the figure looks symmetric (as it should be). Something often seen in figures with theory-laden findings, however, is that one part of the figure is missing (Egger, Smith, Schneider, & Minder, 1997). For instance, if a theory had predicted larger vocabulary sizes in old adults than in young adults and all studies had tested this theory, we could have found that the left lower part of Figure 2 was missing, because these involved small-scale studies turning out “weird” results (not worth publishing). Researchers have developed ways in which funnel plots can be used to get less biased estimates of effect sizes (Anderson, Kelley, & Maxwell, 2017; Duval & Tweedie, 2000). However, such techniques require highly powered studies to see the funnel (i.e., the ones at the top of Figure 2). Notice that such studies are the ones that are “needlessly overpowered” and, hence, not much rewarded in psychology research.

## What can be used then?

Thus far the story has been largely negative (and in this respect mimics many existing papers of power analysis). You need an estimate of effect size to get started, and it is very difficult to get a useful estimate.

There is a way out, however. It starts from the question: What is the typical effect size in psychology or, relatedly, what is the smallest effect size of interest (Lakens, Scheel, & Isager, 2018)? This is nicely summarized in the statistical guidelines of the Psychonomic Society, one of the larger and thoughtful publishers in scientific psychology:

“It is important to address the issue of statistical power. Statistical power refers to the sensitivity of a test to detect hypothetical true effects. Studies with low statistical power often produce ambiguous results. Thus it is highly desirable to have ample statistical power for effects that would be of interest to others and to report a priori power at several effect sizes (not post hoc power) for tests of your main hypotheses. *Best practice is to determine what effects would be interesting (e.g., those that one would consider non-negligible, useful, or theoretically meaningful) and then to test a sufficient number of participants to attain adequate power to detect an effect of that size*.” (https://www.psychonomic.org/page/statisticalguidelines, italics added)

A first source of inspiration about worthwhile effect sizes can be taken from Cohen’s (1962, 1988) writings on statistical power. To help readers, Cohen made a distinction between three types of effect sizes: d = .2 for a small effect size, d = .5 for a medium effect size, and d = .8 for a large effect size. Based on this categorization, we could use the medium effect size of d = .5 as a reasonable estimate of a useful effect size and calculate the required numbers of participants based on this size.

However, in recent years it has become clear that most effect sizes in psychology are smaller than d = .5. Two large-scale replication studies of published findings pointed to an average effect size of d = .4 (Camerer et al., 2018; Open Science Collaboration, 2015). The same value is found in meta-analyses (Bosco, Aguinis, Singh, Field, & Pierce, 2015; Gignac, & Szodorai, 2016; Stanley, Carter, & Doucouliagos, 2018). So, an estimate of d = .4 seems like a better target, even though for some research questions it will still be too high. For instance, in a recent replication study of 28 classic and contemporary published findings, the mean effect size was only d = .15 (compared to d = .6 in the original studies; Klein et al., 2018).

An effect size of d = .4 is further interesting, because it is an effect size that starts having practical relevance. For a repeated-measures factor it means that two thirds of the participants show the effect. For a between-groups factor, it means that you have 61% chance of finding the expected difference if you test a random participant from each sample. An effect size of d = .4 corresponds to a correlation of r = .2.

In the examples below, we will use an effect size of d = .4 as the most reasonable estimate to look for a non-negligible, useful, or theoretically meaningful effect if you have no further good evidence about the effect size.

We further assume that you are interested in a power of 80% to find the effect if it exists at the population level (i.e., that a properly powered study is a study with 80% power). This is the traditional value used, even though it may be considered rather low, as it entails a 20% chance of not finding a theoretically important finding. We will return to this issue in Table 9, when we examine how many extra participants are required to increase the power to 90%.^6^

Finally, in all our examples we assume that you have balanced designs. That is, each cell of the design contains the same number of observations. Unbalanced designs are known to have lower power, the more so when the imbalance becomes stronger. Imbalances are more prevalent for between-groups variables than repeated-measures variables, as participants in the various groups must be recruited, whereas most participants take part in all within conditions. As a rule of thumb, the sum of the participants in the various conditions must be higher than the recommendations given in the present text (on the basis of simulation, some 20% extra seems required for designs that have a 2:1 ratio).

## The easy parts: t-tests and simple correlations

Nearly every text on power deals with the simplest cases: t-tests and single correlations. For t-tests, a distinction is made between a t-test for a between-groups factor and a t-test for a repeated-measures factor (see also under ANOVA).

Below are the numbers you need for a test of p < .05, two-tailed. They can easily be calculated on the basis of software packages such as G*Power (Faul, Erdfelder, Lang, & Buchner, 2007), MorePower (Campbell & Thompson, 2012), or Lenth (2006).^7^

– t test between-groups: Two groups of 100 participants each
– t test repeated-measures: One group of 52 participants
– correlation: 194 data pairs

These numbers are part of the guidelines to be used for more complex designs. As a rule of thumb, never expect the numbers for complex designs to be lower than the numbers for simple designs (see below for the few exceptions). For between-groups designs, assume that the number of participants per condition is the one you have to keep constant (so, a design with three groups will require at least 3 groups of 100 participants).

The number of data pairs for a simple correlation can be extended to multiple regression analysis. If we assume (1) that the correlation between the predictors and the dependent variables is r = .2, (2) that the intercorrelations among the predictors are r = .2 as well, and (3) that we are interested in the impact of the individual predictors rather than the statistical significance of the overall regression analysis, the following rule of thumb seems to be a good approximation for an 80% powered study: 100 participants plus another 100 per predictor variable (Brooks & Barcikowski, 2012; Knofczynski & Mundfrom, 2008; Maxwell, 2000). So, for one predictor you need 200 participants, for 2 predictors you need 300 participants, and so on. The required numbers are higher when the intercorrelations among the predictors are higher than the correlations of the predictors with the dependent variable (Maxwell, 2000).

In recent years, **Bayesian analysis** has been proposed as an alternative to the traditional frequentist tests, such as t-tests and ANOVAs (e.g., Etz & Vandekerckhove, 2018; Kruschke & Liddell, 2018; Rouder, Morey, Verhagen, Swagman, & Wagenmakers, 2017; Rouder, Speckman, Sun, Morey, & Iverson, 2009; Wagenmakers et al., 2018). An advantage of Bayesian analysis is that it gives information about the relative likelihood not only of the alternative hypothesis but also of the null-hypothesis. A Bayesian factor of 10 or more is considered as strong evidence for the alternative hypothesis; a Bayesian factor of .10 or less is considered as strong evidence for the null hypothesis.

It is important to know that Bayesian analysis is a refined approach (Depaoli & van de Schoot, 2017), with the required number of participants depending on choices made. In the analyses below, we use a crude method, recommended for researchers without detailed knowledge of the processes they are investigating. It is implemented as the default procedure in analysis packages such as BayesFactor (Morey, Rouder, Jamil, Urbanek, Forner, & Ly, 2018) and JASP (Wagenmakers et al., 2018). The analysis is based on non-informative JZS priors with medium rescaling ({\rm{rscale}} = {\raise0.5ex\hbox{$\scriptstyle {\sqrt 2 }$}
\kern-0.1em/\kern-0.15em
\lower0.25ex\hbox{$\scriptstyle 2$}}). This represents a lack of knowledge about the values of the parameters being estimated (Rouder, Speckman, Sun, Morey, & Iverson, 2009). It is the analysis likely to be used by researchers unfamiliar with the details of Bayesian analysis, who want to use the technique for null hypothesis significance testing.

The default Bayesian analysis implemented in current software packages requires more participants than traditional frequentist tests with p < .05, an aspect we will return to in the discussion section. There are no power calculators for Bayesian analyses yet, but we can estimate the power of existing algorithms (e.g., the library BayesFactor in R, also used in JASP) via simulation (see https://osf.io/8uaxb/ for the programs we used). Such simulations tell us that we need the following numbers for 80% power (for simulations with large numbers rounded up values are given).

– Bayesian analysis between-groups: Two groups of 190 participants each
– Bayesian analysis repeated-measures: One group of 100 participants
– Bayesian analysis correlation: 370 data pairs

An advantage of Bayesian analysis is that it allows you to conclude in favor of the null hypothesis. What is usually not mentioned is that you need many data for that. On the basis of simulation, the following are the numbers for BF < .1:

– Bayesian analysis null effect between-groups: Two groups of 1,200 participants each
– Bayesian analysis null effect repeated-measures: One group of 720 participants
– Bayesian analysis null effect correlation: 3,000 data pairs

Because Bayes factors of .10 look unreachable for most research, it may be good to also include the numbers for Bayes factors smaller than 1/3 (considered as moderate evidence for the null hypothesis). They are more feasible, as can be seen below.

– Bayesian analysis null effect between-groups: Two groups of 110 participants each
– Bayesian analysis null effect repeated-measures: One group of 60 participants
– Bayesian analysis null effect correlation: 250 data pairs

Because the numbers are lower than those for the alternative hypothesis, this means that the participant numbers for the alternative hypothesis can be used to simultaneously measure strong evidence for the alternative hypothesis (BF > 10) and moderate evidence for the null hypothesis (BF < .33).

A typical mistake made within traditional statistical testing is that the absence of a significant effect is interpreted as evidence for the null hypothesis. This is wrong, because only some of the non-significant effects are due to the null hypothesis being true. To show that the data are in line with the null hypothesis, you must go further and demonstrate that the effect is so small that it does not have theoretical importance (Lakens et al., 2018). This can be done by examining whether the effect remains within two pre-established narrow borders around zero. For instance, we could say that evidence for the null-hypothesis is strong enough when the obtained effect is significantly larger than d = –.2 and smaller than d = .2. This test is known as the two one-sided tests (TOST) procedure and R has a package TOSTER to run it (Lakens et al., 2018). With α = .05, the numbers we get are shown below. For correlations we assume that the lower and upper limits are r = –.1 and r = +.1.

– TOST null effect between-groups: Two groups of 430 participants each
– TOST null effect related pairs: One group of 215 participants
– TOST null effect correlation: 860 data pairs

The simple tests just described are the backbone of power analysis, because we use them every time we calculate post hoc pairwise comparisons to understand the pattern of effects observed in more complicated designs. If anything, these post hoc tests will require more participants than the numbers reported above because they need to be corrected for multiple testing (e.g., by using the Bonferroni correction). On the positive side, if we have specific predictions for pairwise comparisons, we can use one-tailed tests, which reduce the numbers of participants needed. For instance, a one-tailed repeated-measures t test (p < .05, .8 power) requires only 41 participants for d = .4 instead of 52; a one-tailed between-groups t test (p < .05, .8 power) requires only two groups of 78 participants each.

## One-way ANOVA with three between-groups levels

In terms of power, simple designs (one independent variable, two levels) are preferable and researchers are advised to keep their designs as simple as possible. However, sometimes it makes sense to have three levels of a categorical variable. This is the case, for instance, when there is a known difference between two conditions, and a third condition is examined which is expected to yield results in line with one of the two conditions, or results in-between. Then it makes sense to compare the new condition to the two already well-established conditions. For example, it is known that associated words prime each other. Associated words are words that spontaneously come to mind upon seeing a prime word. Examples are boy after seeing girl, warm after seeing cold, bread after seeing butter, and so on (see De Deyne, Navarro, Perfors, Brysbaert, & Storms, 2019, for association data on 12,000 English words). The second word (the target) is processed faster when it follows the first word (prime) than when it follows an unrelated word (*girl-boy* vs. *card-boy*). Suppose a researcher now wants to know to what extent, non-associated, semantically related words prime target words (e.g., *mum-boy*). Then it makes sense to present the three types of primes in a single experiment to (a) make sure that a priming effect is observed for the associated pair, and (b) to examine how large the priming effect is for the new primes relative to the associated pairs.

The semantic priming example is likely to be a repeated-measures experiment. However, the same reasoning applies to a between-groups design. We start with the latter design (**three independent groups**), because the independence of observations makes the calculations easier. In line with the previous analyses, we assume that the standardized effect size between the two extreme conditions is d = .4. There are two scenarios of interest: (1) the new condition is in line with one of the existing conditions (either the lower or the higher) and has an effect size of d = .4 with the other condition, or (2) the new condition is midway in-between the other two conditions (i.e., differs d = .2 from each condition).

For such an experiment, it is not enough to have a significant effect in the omnibus ANOVA. It is also important to be able to find the population pattern in post hoc tests (why else include three conditions). So, we need to know how many observations we require for p < .05 in the omnibus ANOVA; and p < .05 one-tailed t tests, for the expected differences in three post hoc tests with Bonferroni correction. Simulations indicate the following numbers.

– New condition similar to one of the other conditions: Three groups of 145 participants each
– New condition midway in-between the two other conditions: Three groups of 580 participants each

The numbers are especially high in the last design because of the need for significant post-hoc tests. You need two groups of 443 participants for a t-test of independent groups with d = .2, alpha = .033,^8^ and power = .8. The present situation is even more restrictive, because you need a significant effect in the omnibus test and three significant post-hoc t-tests (with Bonferroni correction) of which two address effect sizes of d = .2 and one an effect size of d = .4. This illustrates once again that you must have good reasons to add extra conditions to your design!

To illustrate how people can misread power requirements, we can have a look at what G*Power recommends for an ANOVA F-test (fixed effects, omnibus, one-way). For such a test, G*Power needs the effect size expressed as f-coefficient. The f-coefficient is roughly the square root of the better known (partial) eta squared value, and for a pairwise between-groups comparison f = d/2. When we select effect size f = .2 (equal to d = .4), alpha = .05, power = .8, and two groups, we get the 100 participants per group also required for the t-test with unrelated samples. When we increase the number of groups to 3, G-Power informs us that we now only need 246 participants or 82 per group (Figure 3). If we run the simulation with these numbers, we find that the omnibus ANOVA is significant 75% of the times but that the complete pattern is present in only 49% of the samples. The reason why the omnibus test is not significant 80% of the time is that the introduction of a third condition slightly lowers the f-value, which we should have taken into account.^9^

**Figure 3:**
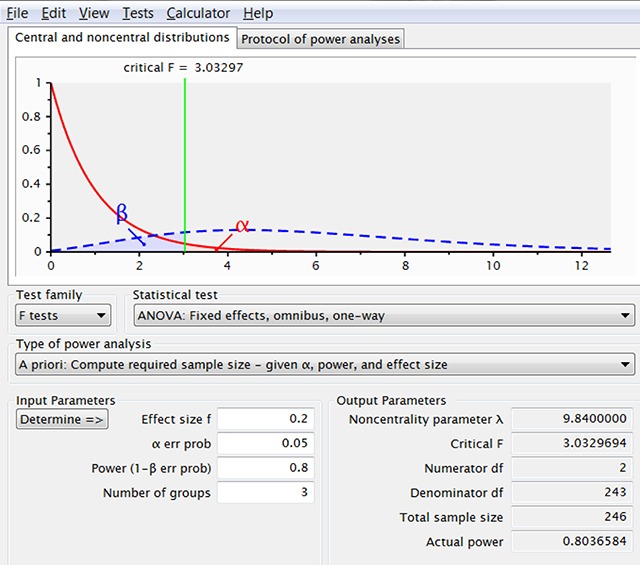
Output of G*Power when we ask the required sample sizes for f = .2 and three independent groups. This number is an underestimate because the f-value for a design with d = .4 between the extreme conditions and a smaller effect size for the in-between condition is slightly lower than f = .2. In addition, this is only the power of the omnibus ANOVA test with no guarantee that the population pattern will be observed in pairwise comparisons of the sample data.

We can also run Bayesian analysis for a design with three independent groups. We want a Bayes factor > 10 for the omnibus ANOVA and each of the pairwise comparisons expected to be significant, and BF < 3 for the comparison not supposed to differ. In Bayesian analysis, the Bayes factors have been argued not to require adjustment for multiple tests (e.g., Kruschke & Liddell, 2018). This is unlikely to be true but as a first approximation can do (see de Jong, Marsman, & Wagenmakers, 2019, for further ideas).

– New condition similar to one of the other conditions: Three groups of 230 participants each
– New condition midway in-between the two other conditions: Three groups of 950 participants each

## One-way repeated-measures ANOVA with three levels

Because the numbers of participants in properly powered between-groups designs are so high, many researchers are motivated to run designs within participants, also called repeated-measures designs. Unfortunately, different effect sizes can be calculated for repeated-measures designs and this is the source of much confusion and incorrect use of power calculators.

To illustrate the issue, we make use of a toy dataset, shown in Table 2. It includes the average reaction times (in milliseconds) of 10 participants to target words preceded by related primes and unrelated primes. Every participant responded to both conditions. So, the manipulation is within subjects.

**Table 2:** Example of data (reaction times) from a word recognition experiment as a function of prime type (related, unrelated).

Participant	Related	Unrelated	Priming

p1	638	654	16
p2	701	751	50
p3	597	623	26
p4	640	641	1
p5	756	760	4
p6	589	613	24
p7	635	665	30
p8	678	701	23
p9	659	668	9
p10	597	584	–13
**Mean**	**649**	**666**	**17**
**Standard dev.**	**52.2**	**57.2**	**17.7**

As we can see in Table 2, almost all participants showed the expected priming effect (faster responses after a related prime than after an unrelated prime). Only the last participant had a difference in the opposite direction.

The **effect size d in a t-test for related samples** is based on the difference scores. You can simply calculate it by dividing the mean of the difference scores by their standard deviation: d = 17/17.7 = .96. Notice that the effect size is uncommonly large, as often happens in statistics textbooks when small datasets are used as examples.^10^ As it happens, the t test is significant for Table 2: t(9) = 3.04, p = .014. We can also calculate d on the basis of t with the equation d = {\textstyle{t \over {\sqrt N}}} = {\textstyle{{3.04} \over {3.16}}} = .96.

The d-value for a t test is the one we implicitly assume when we think of a pairwise effect size in a repeated-measures design. However, it is not straightforward to get this value from an ANOVA analysis. Most of us would probably use partial eta squared (η^2^_p_) as the initial estimate of the effect size, because this is given by most software packages. If we run an ANOVA on the data of Table 2, we get F(1,9) = 9.24, p = .014, η^2^_p_ = .507.

One way in which we may go wrong in estimating d on the basis of η^2^_p_ is that we use the often quoted conversion equation from η^2^_p_ to d:

d \approx 2*\sqrt {\frac{{\eta _p^2}}{{1 - \eta _p^2}}}\approx 2*\sqrt {\frac{{.507}}{{1 - .507}}}  = 2.03.

Unfortunately, this equation is only valid for between-groups. For repeated-measures the correct equation is:

d \approx \sqrt {\frac{{\eta _p^2}}{{1 - \eta _p^2}}} = \sqrt {\frac{{.507}}{{1 - .507}}}  = 1.01.

11

The multiplication by 2 is not warranted because the two observations per participant are not independent (as can be seen in the degrees of freedom in the t test). As a result, the typical conversion overestimates the d-value by a factor of two. Still, the error is easily made because people use the same equation for between-subjects designs and within-subjects designs.

Another way in which we can go astray with the calculation of d in repeated-measures designs is due to the fact that d can be defined in two ways. First, it can be defined as we just did on the basis of difference scores, and this definition is the one that matters for power analysis. However, d can also be defined as the difference in means divided by the mean standard deviation. Then it amounts to d ≈ 17/[(52.2 + 57.2)/2] = .31 (instead of d = .96). The latter definition is interesting for meta-analysis because it makes the effect size comparable in between-groups designs and repeated-measures designs. Suppose, for instance, we want to make a meta-analysis of all studies comparing reading speed in silence and reading speed under noise (Vasilev, Kirkby, & Angele, 2018). Some of the studies included in the meta-analysis have the same participants in both conditions; other studies have different participants in the conditions. Using the d-values of the t tests for related and unrelated samples would give higher d-values for related samples than for unrelated samples even though the differences in reading speed may be the same (compare our values of d = .96 and d = .31 for the data in Table 2).

Because there are two definitions of d for pairwise comparisons in repeated-measures designs, it makes sense to give them different names and to calculate both. The d-value based on the t-test for related samples is traditionally called d_z_, and the d-value based on the means d_av_ (e.g., Lakens, 2013). For the example of Table 2, d_z_ = .96 and d_av_ = .31.

Because d_z_ and d_av_ are related to each other, we can derive the mathematical relation between them. The crucial variable is the correlation of the repeated-measures. If we correlate the response times in the related and the unrelated condition across participants in Table 2, we find a surprising correlation of r = .95 (N = 10). This is because all participants show more or less the same difference score, despite large differences in overall response times (going from 590 ms to 760 ms).

More specifically, it can be shown that (e.g., Morris & DeShon, 2002):

{d_z} = \frac{{{d_{av}}}}{{\sqrt {2\left({1 - {r_{XY}}} \right)}}} = \frac{{.31}}{{\sqrt {2*\left({1 - .95} \right)}}} \approx .96.

The inclusion of the correlation in the equation makes sense, because the more correlated the observations are across participants, the more stable the difference scores are and, hence, the larger d_z_. The inclusion of the correlation in the equation is also the reason why power calculators such as G*Power ask for the correlation between the conditions when the power for repeated-measures ANOVAs is estimated (usually to the bewilderment of uninformed users).

The equation is further interesting because it says that d_z_ = d_av_ when the correlation between the two conditions is r = .5, and that d_av_ is larger than d_z_ when r < .5 (which is the case in between-groups designs, where no correlation is expected among data pairs of the two conditions).

We can easily see how things can go wrong in using a power calculator. If a user assumes an effect size of d = .4 and a correlation of .95 between the conditions, the calculator informs them that 8 participants are enough to reach 80% power for alpha = .05, as shown in Figure 4. This is the same number we would obtain if we entered d = {\textstyle{{.4} \over {\sqrt {2\left({1 - .95} \right)}}}} = 1.265 in a t test for related samples. In contrast, if we use a correlation of r = .5, the calculator rightly informs us that we need 52 participants for an 80% powered experiment, in line with the t test for repeated-measures.

**Figure 4:**
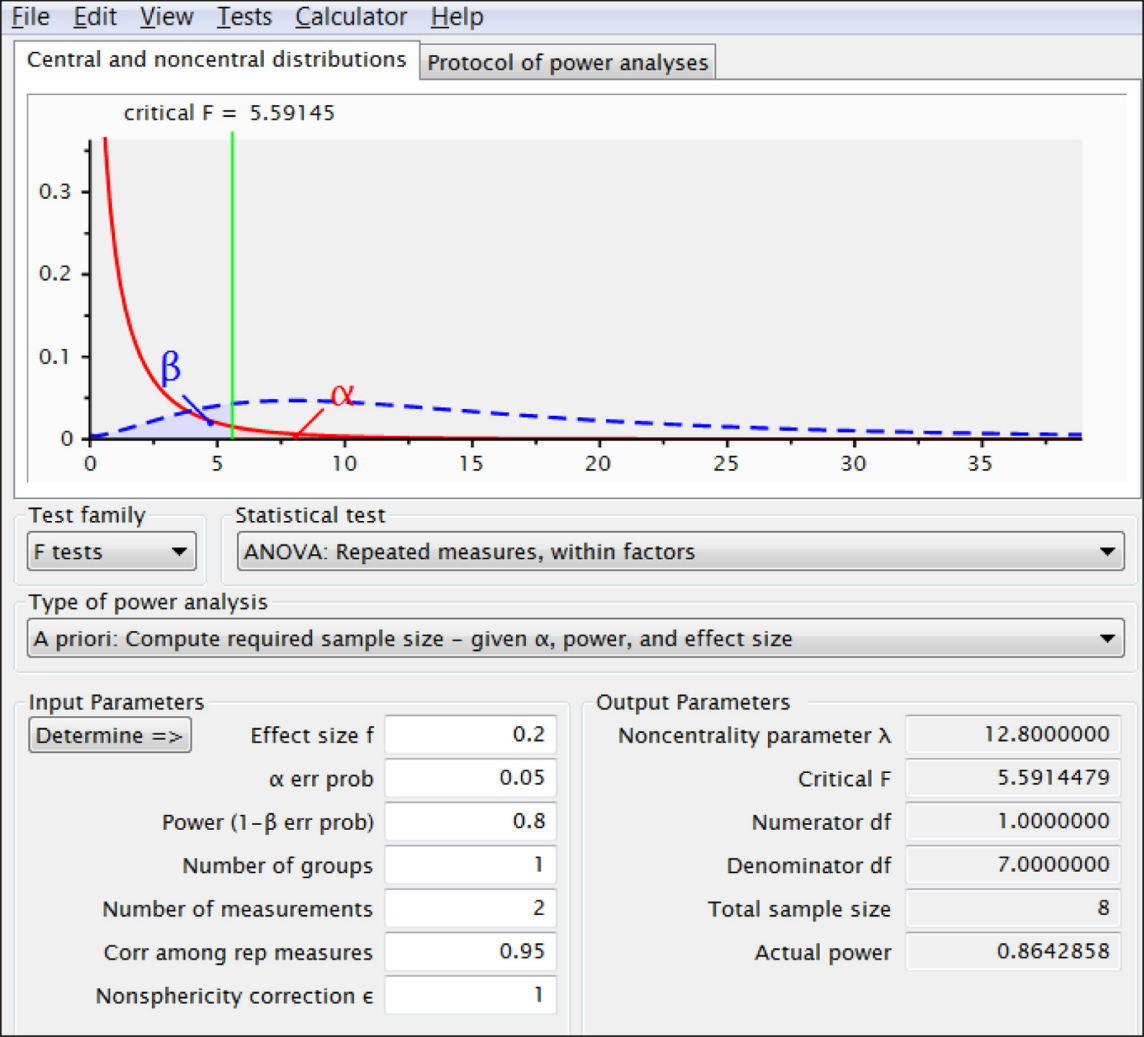
If one increases the correlation among the repeated measurements, G*Power indicates that fewer observations are needed. This is because G*Power takes the effect size to be d_av_, whereas the user often assumes it is d_z_.

It might look like the correlation of .95 between the repeated-measures in Table 2 is unrealistically high. However, it is a value that can be observed in well-run reaction time experiments. To have a better idea of the correlations observed in psychological research, we analyzed some of the repeated-measures experiments studied in two large replication projects: Camerer et al., 2018 (available at http://www.socialsciencesreplicationproject.com/) and Zwaan, Pecher, Paolacci, Bouwmeester, Verkoeijen, Dijkstra, & Zeelenberg, 2018 (available at https://osf.io/shej3/wiki/home/). The advantage of replication studies is that the full datasets are available. Table 3 shows the results. In particular studies with reaction times and ratings (two heavily used dependent variables in psychological research) have high intercorrelations between the levels of a repeated-measures factor.^12^ The correlations are lower for memory accuracy. We will return to these correlations in the second half of the article.

**Table 3:** Correlations observed between the levels of a repeated-measures factor in a number of studies with different dependent variables.

Study	Dependent variable	Correlation

*Camerer et al. (2018)*		
Aviezer et al. (2012)	Valence ratings	–0.85
Duncan et al. (2012)	Similarity identification	0.89
Kovacs et al. (2010)	Reaction time to visual stimuli	0.84
Sparrow et al. (2011)	Reaction time to visual stimuli	0.81
*Zwaan et al. (2018)*		
associative priming	Reaction time to visual stimuli (Session 1)	0.89
	Reaction time to visual stimuli (Session 2)	0.93
false memories	Correct related-unrelated lures (Session 1)	–0.47
	Correct related-unrelated lures (Session 2)	–0.14
flanker task	RT stimulus congruent incongruent (Session 1)	0.95
	RT stimulus congruent incongruent (Session 2)	0.93
shape simulation	RT to shape matching sentence (Seesion 1)	0.89
	RT to shape matching sentence (Seesion 2)	0.92
spacing effect	Memory of massed v. spaced items (Session 1)	0.35
	Memory of massed v. spaced items (Session 2)	0.55

There are two surprising cases of negative correlations in Table 3. The first is a study of valence ratings (from negative to positive on a Likert scale from 1 to 9). Apparently, the participants who rated the positive images very positively also rated the negative images very negatively, whereas other participants had less extreme ratings. The second negative correlation comes from a study comparing memories for information not presented (false memories) to memories for information presented. Apparently, participants who remembered less had a tendency to report more false memories.

Just like for between-groups designs, G*Power suggests that the number of required participants decreases as more repeated-measures conditions are added. For f = .2, 2 levels, alpha = .05, power = .8, the required number is 52 (as in the t test). For three levels, it becomes 42; for four levels 36; and for five levels 32. This is because we (wrongly) assume that the f-value does not decrease as more levels with in-between values are added, and because we are only asking for the significance of the omnibus ANOVA test. This is where simulations form a nice addition.

There are two ways in which we can simulate the design. First we make the correlation between the repeated-measures equal to r = .50. Then we know that d_z_ = d_av_. In that situation (r = .50; d_av_ = .4; p < .05 in the omnibus analysis; significant one-tailed Bonferroni corrected post hoc t tests for the pairwise comparisons that are different at the population level), we see that the following numbers of participants are required:

– New condition similar to one of the other conditions: 75 participants
– New condition midway in-between the two other conditions: 290 participants

The second way in which we can simulate the data is to assume a correlation of r = .90 and adapt d_av_ such that d_z_ stays at .4. We do this by recoding d_av_ as {d_{av}} = {d_z}*\sqrt {2\left({1 - {r_{XY}}} \right)} or d_av_ = .4 * .45 = .18. The required participant numbers should remain more or less the same as for the simulations with r = .5, as they indeed do:

– New condition similar to one of the other conditions: 75 participants
– New condition midway in-between the two other conditions: 290 participants

Again notice that the addition of an extra condition does not decrease the number of participants required if we want to correctly interpret the data, contrary to what an uninformed use of G*Power suggests. As a matter of fact, adding a condition increases the number of participants to be tested, even in a repeated-measures design. This is not because the omnibus ANOVA fails to reach significance (as it happens, it is seriously overpowered with the numbers recommended), but because many observations are needed to replicate the pattern of pairwise population differences that drive the interaction.

For a Bayesian analysis (BF > 10 in the omnibus ANOVA and the relevant post hoc tests, BF < 3 for the non-significant pairwise comparisons), these are the numbers we require:

Repeated-measures variable r = .50

– New condition similar to one of the other conditions: 120 participants
– New condition midway in-between the two other conditions: 540 participants

Repeated-measures variable r = .90, {d_{av}} = {d_z}*\sqrt {2\left({1 - {r_{XY}}} \right)}  = .4*.45 = .18.

– New condition similar to one of the other conditions: 125 participants
– New condition midway in-between the two other conditions: 540 participants

For the remainder of the analyses, we always tested whether we obtained the same results for r = .50 and r = .90 in the simulations (as it should). Because this was the case, we no longer report the separate results. Just know that the numbers are valid for correlations from .5 to .9 (and beyond) between the levels of a repeated-measures variable.

## Two-way ANOVA with repeated-measures

Sometimes we want to include two variables in our design, for instance two repeated-measures factors. We only discuss a 2 × 2 design, as this is the most common design (one needs good reasons – and many participants – to go for designs with more levels).

There are two reasons why we may want to include an extra variable in a study. The first is that we want to control for a possible nuisance variable. In that case we are primarily interested in the main effect of the target variable. We do not expect the other variable to have much effect, but we include it for certainty. So, we assume that factor A has d = .4 and factor B has d = .0 and also does not interact with factor A. Basically, the question is the same as in a t-test for repeated-measures. The only things that differ are that the design has become more complex and that we collect four observations from each participant instead of two.

Repeated-measures variables A (d_z_ = .4) and B (d_z_ = .0) no interaction:

– F-test (p < .05): 27 participants
– Bayesian test (BF > =10): 52 participants

The number of required participants is about half that of the t test for related samples. This is because the effect of A is observed across both levels of B and we have twice as many observations per participant (four instead of two).

A mistake often made in this respect, however, is that researchers assume that the reduction of participants remains valid when they have 80 stimuli (e.g., target words preceded by related or unrelated primes) and for the t test have two conditions with 40 stimuli per condition, whereas for the 2 × 2 ANOVA they have 4 conditions and 20 stimuli per condition. In such a situation, the number of observations stays the same and so the number of participants for the 2 × 2 ANOVA must be higher (roughly equal to the t test). We will return to the issue of multiple observations per cell later in the article (see also Brysbaert & Stevens, 2018).

The second reason why we may want to include two variables in the design is that we are interested in the interaction between the variables. There are three possible scenarios (Figure 3). In the first case we expect a full crossing of the effects. That is, for one level of factor A the effect of factor B will be positive, and for the other level of A it will be negative (left panel of Figure 3). The most common origin of such an interaction is when a control variable has an effect as strong as the variable we are interested in. For instance, we have to use different stimulus lists for a memory experiment and it turns out that some lists are considerably easier than the other. Then we are likely to find a cross-over interaction between the variable we are interested in and the lists used in the different conditions. Ironically, this interaction is the easiest to find. In such a scenario the numbers required are the same as for the main effects we just saw (Perugini, Gallucci, & Costantini, 2018).

**Figure 3:**
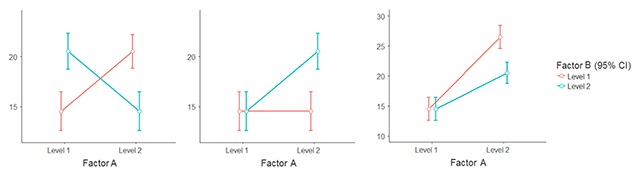
Different types of interactions researchers may be interested in. Left panel: fully crossed interaction. Middle panel: the effect of A is only present for one level of B. Right panel: The effect of A is very strong for one level of B and only half as strong for the other level.

When the two repeated-measures factors are of interest, a more likely scenario is one in which we expect an effect of Factor A (d = .4) for one level of factor B but not for the other. The power requirements of this type of interaction have long been misunderstood. The issue is that in such a case the interaction effect does not have d = .4 but d = .2 (the average of .4 and .0). Needless to say, this reduction of effect size has serious consequences for the number of participants required to find the interaction. Simonsohn (2014) showed that the numbers have to be multiplied by four (see also Giner-Sorolla, 2018; Perugini et al., 2018).

In addition, we not only want to find a significant interaction. We also want to find the significant effect of A at the appropriate level of B in a separate pairwise test, and the absence of the effect at the other level. Simulations indicate that we have 80% chance of finding this constellation when we have the following numbers:

– F test (interaction in omnibus test p < .05; post-hoc t-test for B level with difference in A p < .10/2 [one-tailed, Bonferroni corrected]; post-hoc one-tailed t test level B without difference in A p > 10/2): 110 participants
– Bayes test (interaction BF > 10, BFs post-hoc tests >10 and <3 respectively): 210 participants

As argued by Simonsohn (2014), this is indeed a multiplication of the number of participants by four (27 * 4 ≈ 110, 52 * 4 ≈ 210).

Finally, the third scenario is one in which we have a very robust effect (d = .8) but we think the effect will be smaller if something is changed. Indeed, much scientific research examines the boundary conditions of well-established effects.

Before we look at the number of participants required to reliably find such a pattern, it may be good to know that this type of interaction is often uninterpretable (Garcia-Marques, Garcia-Marques, & Brauer, 2014). It is only interpretable when the size of the interaction is larger than the smallest main effect. When the size of the interaction is equal to the smallest main effect (as in the right panel of Figure 3), the interaction is borderline interpretable. However, when the size of the interaction is smaller than the smallest main effect, it cannot be interpreted, because the interaction could be due to a non-linear relationship between the unobservable process of interest and the overt response that can be measured. Garcia-Marques et al. (2014) argue that the requirement of non-minimal size should be kept in mind when setting up a study: If the interaction is your prime interest, you must make sure that it is stronger than at least one of the main effects. More in general, Garcia-Marques et al. (2014) showed that in a 2 × 2 design the effect with the smallest size (Factor A, Factor B, or the interaction) cannot be interpreted. As a rule of thumb, the interaction will not be smaller than both main effects when the lines touch or cross each other at some point.^13^

These are the numbers of participants required for 80% power:

– F test (interaction in omnibus test p < .05; post-hoc t-test for B level with difference in A p < .10/2 [one-tailed, Bonferroni corrected]; post-hoc one-tailed t test level B without difference in A p < .10/2): 105 participants
– Bayesian test (interaction BF > 10, BFs post-hoc tests >10): 200 participants

The numbers of participants required are very similar to the situation depicted in the middle panel of Figure 3. This is how it should be because the right panel of Figure 3 can be thought of as the middle panel with an additional main effect of d = .4 for A. So, the interaction is the same. The remaining small difference in numbers is due to the extra requirements related to the pairwise post hoc tests.

## Two-way ANOVA with one repeated-measures factor and one between-groups factor

When performance of two groups is compared, researchers often use a so-called split-plot design with one between-groups variable and one repeated-measures factor. Indeed, researchers often wonder whether such a design is not more powerful than a simple between-groups comparison. Suppose you want to examine whether students with dyslexia are disadvantaged in naming pictures of objects. What is to be preferred then? Use a simple one-way design in which you compare students with dyslexia and controls on picture naming? Or use a 2 × 2 design in which you ask both groups of participants to name pictures and to read words (for which you know there will be a difference between the groups)? For a long time, the author was convinced that the latter option was to be preferred (because of what power calculators told me), but is this confirmed in simulations?

Before we start with the interactions, it is good to have a look at the main effect of the repeated-measures factor. Often Latin-square designs are used to counterbalance stimuli across conditions (Pollatsek & Well, 1995). In a first scenario, the between-groups variable is not expected to have a main effect or to interact with the repeated-measures factor. It just increases the complexity of the design. For this scenario, the following are the numbers to attain 80% power for the main effect of the repeated-measures variable equal to d = .4.

- F-test (p < .05): two groups of 27 participants each
- Bayesian analysis (BF > 10): two groups of 50 participants each

In a second scenario, the Latin-square group interacts with the main effect of the repeated-measures variable. One stimulus set is easier than the other, and this introduces an effect of equal size. So, for one group of participants the difference between conditions is d = .8; for the other group it is d = .0. How many participants do we need in such a scenario to find a main effect of the repeated-measures variable with a power of .80?

- F-test (p < .05): two groups of 27 participants each
- Bayesian analysis (BF > 10): two groups of 50 participants each

This is interesting news, because it tells us that we can add extra between-groups control factors to our design, without having much impact on the power to detect the main effect of the repeated-measures variable, as was indeed argued by Pollatsek and Well (1995).

We can also look at the power of the between-groups variable. Is it the same as for the between-groups t test, or does the fact that we have two observations per participant make a difference? And does the outcome depend on the correlation between the levels of the repeated-measures variable? Here are the data:

- F-test between-groups variable (p < .05):
  - r_repeated measure_ = .50: two groups of 75 participants each
  - r_repeated measure_ = .90: two groups of 95 participants each
- Bayesian analysis (BF > 10)
  - r_repeated measure_ = .50: two groups of 145 participants each
  - r_repeated measure_ = .90: two groups of 180 participants each

The lower the correlation between the levels of the repeated-measures variable, the smaller the number of participants becomes. This can be understood, because highly correlated data do not add much new information and they do not much decrease the noise in the data. In contrast, uncorrelated data add new information.

When the interaction is the focus of attention, we have to make a distinction between the three types of interactions illustrated in Figure 3. The fully crossed interaction is most likely to be found with control variables (e.g., counterbalancing stimulus lists over conditions by making use of Latin-square groups; Pollatsek & Well, 1995). The other two interactions are more likely to be of theoretical interest. These are the numbers needed for proper power (80%).

Opposite effects in the two groups; cross-over interaction (d = +.4 and d = –4):

- F-test (p < .05)
  - Only interaction significant: two groups of 27 participants each
  - Interaction plus two post-hoc tests significant: two groups of 67 participants each
- Bayesian analysis (BF > 10)
  - Only interaction significant: two groups of 50 participants each
  - Interaction plus two post-hoc tests significant: two groups of 125 participants each

If we only look at the significance of the interaction, then two groups of 27 participants each are enough for an F-test. Half of the time, however, the interaction will not be accompanied by the right pattern of post-hoc effects in the groups. For the complete pattern to be present, we need two groups of 67 participants for the F-test and two groups of 125 participants for the Bayesian analysis.

Effect in one group (d = .4) not in the other:

- F-test (interaction significant at p < .05, plus significant one-tailed Bonferroni corrected main effect in one group, not in the other): two groups of 100 participants each
- Bayesian analysis (BF > 10 for interaction and main effect in the expected group; BF < 3 for main effect in the other group): two groups of 195 participants each

Interestingly, the numbers of participants required in these 2 × 2 split-plot designs are very much the same as the numbers required for a between-groups one-way design with d = .4. So, a split-plot design is not more powerful than a between-subjects design in terms of participants required. It does give more information, though, because it adds information about a possible main effect of the between-groups variable, and the group dependency of the repeated-measures effect.

The same numbers are needed for a situation in which a large effect is observed in one group (d = .8) and a smaller effect in the other group (d = .4), as can be expected given that it is the same interaction with an extra d = .4 main effect for the repeated-measures variable.

Notice how different the outcome is from the conviction mentioned in the introduction that you can find small effect sizes in a split-plot design with 15 participants per group. Still, the authors were not lying, because you can get such a small number from G*Power, when you introduce some reasonable conditions, as can be seen in Figure 4.

**Figure 4:**
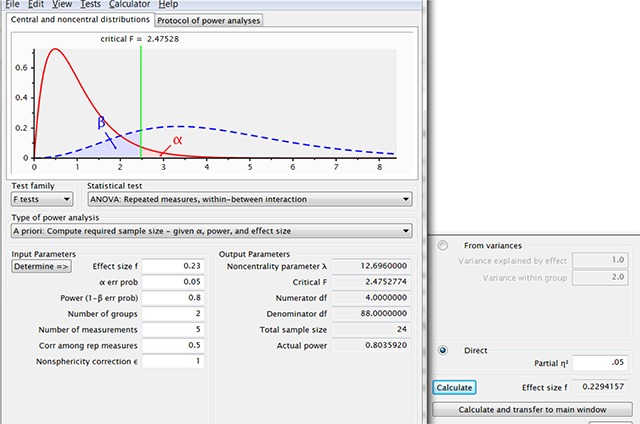
Figure illustrating how you can convince yourself with G*Power that two groups of 15 participants allow you to find effect sizes of f = .23 in a split-plot design with two groups and five levels of the repeated-measures variable. When seeing this type of output, it good to keep in mind that you need 50 participants for a typical effect in a t-test with related samples. This not only sounds too good to be true, it is also too good to be true.

To understand the number of participants advised by G*Power in Figure 4, it is important to know that the number is only valid under the following conditions: (1) the interaction effect is as big as the main effect (i.e., the equivalent of a fully crossed interaction), (2) you compare designs in which you only have one observation per condition (so, more levels of the repeated measure gives you more information), and (3) you are only interested in the overall interaction effect of the omnibus test, not whether the pattern of pairwise differences agrees with what is in the population.

Some authors have recommended using an analysis of covariance for a situation in which pretest and posttest scores of two groups of people are compared (e.g., Dimitrov & Rumrill, 2003; Egbewale, Lewis, & Sim, 2014; O’Connell, Dai, Jiang, Speiser, & Ward, 2017). Rather than analyze the data with a 2 × 2 design (two groups and two measurement times), the suggestion is to analyze the posttest data with a one-way ANCOVA in which the group is the only independent variable and the pretest scores are used as covariate. To check whether this analysis is more powerful, we ran simulations to determine the minimum number of participants required.

The scores of Group 2 are d_z_ = .4 higher on second measurement than on first measurement; Group 1 stays the same; both groups are the same on first measurement (i.e., the middle panel of Figure 3):

- F-test group (p < .05)
  - r_repeated measure_ = .50: two groups of 80 participants each
  - r_repeated measure_ = .90: two groups of 100 participants each
- Bayesian analysis (BF>10 for difference between-groups)
  - r = .50: two groups of 150 participants each
  - r = .90: two groups of 190 participants each

The analysis of covariance is indeed more powerful than the 2 × 2 split-plot design, as long as the correlation between the two levels of the repeated measure is lower than .95. At the same time, the power is not increased to such an extent that a researcher can observe typical effect sizes with groups of 25 participants (as erroneously assumed by a researchers mentioned in the introduction).

## Increasing the power by having multiple observations per condition per participant

The number of participants required for an 80% powered studies often surprises cognitive psychologists, because in their experience replicable research can be done with smaller groups. Indeed, samples of 20–24 participants for a long time were the norm in experimental psychology. There are two reasons for this. The first is the illusion of sufficient power based on significant p values, as explained in the introduction (unreplicable studies are a problem in experimental psychology too).

The second reason, however, is that some effect sizes can be brought to d > .4 without using questionable research practices.

The secret lies in the equation {d_z} = {\textstyle{{{d_{av}}} \over {\sqrt {2\left({1 - {r_{XY}}} \right)}}}}.

By increasing the correlation between the two levels of the repeated measure, you can increase d_z_ relative to d_av_. The effect sizes reported in meta-analyses often are d_av_ or a mixture of d_av_ an d_z_. D_av_ is preferred for meta-analysis because it allows researchers to compare results from between-groups designs and repeated-measures designs. However, it cannot always be calculated because the authors of the original studies do not provide enough information in their articles. So, in all likelihood d_z_ values are often included in meta-analyses as well. Nevertheless, the average value of d = .4 found in psychology research is likely to be related more to d_av_ than to d_z_. Still, d_z_ is the value that matters for power analysis. Because of the equation, d_z_ will be 1.5 times d_av_ when r_XY_ = .78 and d_z_ will be twice d_av_ when r_XY_ = .88.

The correlation between two variables depends on the reliability of the variables: Noisy variables with low reliabilities do not correlate much with each other, because they do not even correlate much with themselves. So, by increasing the reliability of the measurements, we can increase d_z_ in a repeated-measures design.

Most cognitive researchers have an intuitive understanding of the requirement for reliable measurements, because they rarely rely on a single observation per participant per condition. A perception psychologist investigating the relationship between stimulus intensity and response speed is unlikely to have each participant respond to each stimulus intensity only once. Instead, they will ask the participant to respond say 40 times to every stimulus intensity and take the average reaction time. Similarly, a psycholinguist studying the impact of a word variable (say, concreteness) on word recognition is unlikely to present a single concrete and abstract word to each participant. Instead, they will present some 40 concrete words and 40 abstract words, also because they want to generalize the findings across stimuli.

Brysbaert and Stevens (2018) showed how a mere 16 ms priming effect (d_av_ = .08) can be turned into a large effect size (d_z_ = .9) when 210 words are presented per condition. Similarly, Zwaan et al. (2018) showed that several effects in cognitive psychology have effect sizes of d_z_ > .5 when based on multiple observations per condition. So, another way to increase the power of the design is not to increase the number of participants but the number of observations per cell of the design (see also Rouder & Haaf, 2018).

Decreasing the noise by averaging per participant and per condition over multiple observations has a second advantage: It decreases the variance within the conditions. This is also true for between-groups studies. Averaging over multiple observations per participants is likely to decrease the interindividual differences within the groups.

The advantage of having several observations per participant per condition was already visible in the designs covered, when we saw that fewer participants are needed for main effects in a 2 × 2 repeated-measures design than for the main effect in a design with a single variable of two levels. Also in the split-plot design, fewer participants were needed than in the corresponding t-test because there was more than one observation per person (at least if the levels of the repeated-measures did not correlate too much, so that there was room for noise).

All in all, it can be expected that averaging across multiple observations per condition per participant will increase the power of an experiment when the following two conditions are met:

1. There is an effect at the population level and every participant is expected to show the effect to some extent (Rouder & Haaf, 2018).
2. There is noise in participants’ responses that can be reduced by averaging.

The latter condition is particularly true for reaction times, where there are huge differences in response speed when the same participant responds several times to the same stimulus. However, noise may be much more limited when participants are asked to rate stimuli. They may give more or less the same ratings to the same items, so that there is no point in repeating items many times.

The easiest way to find out whether profit can be made by increasing the number of responses is to look at the reliability of the dependent variable. Reliability is a cornerstone of correlational psychology and it is a shame that its importance has been lost in experimental psychology (Cronbach, 1957). In the next sections, we will see how the reliability of the dependent variable can be used to optimize the effect size and in that way reduce the number of participants that must be tested.

## Intraclass correlation based on mixed effects modeling is an easy and versatile way to measure the reliability of a dependent variable

When we talk about reliability of a dependent variable, we mean that participants’ scores remain more or less the same when they are tested more than once. Multiple observations can be realized by presenting the same stimulus more than once (e.g., a light flash of a certain intensity) or by presenting several equivalent stimuli (e.g., related questions in a text comprehension study). Table 4 gives an example in which 6 participants responded 4 times to the same stimulus.

**Table 4:** Responses of six participants (P1–P6) to four repetitions of the same stimulus (S1–S4).

	S1	S2	S3	S4

**P1**	7	5	10	6
**P2**	9	11	8	10
**P3**	12	9	17	14
**P4**	11	8	6	18
**P5**	7	3	3	6
**P6**	10	14	7	14

There are several ways to calculate the reliability of the data in Table 4. For instance, we could calculate the correlation between S1 and S2. This is r = .59. We could also calculate the correlation between the average of the first two presentations and the average of the second two presentations. This correlation is known as the split-half correlation. For Table 4 it amounts to .74.

Shrout and Fleiss (1979) showed how to calculate two summary measures of reliability, which they called intraclass correlations. The first measure corresponds to the average correlation between the repetitions. The second corresponds to the expected correlation between the mean scores of the four repetitions and the mean scores that can be expected if another four repetitions were run. Brown and Spearman in the beginning of the 20^th^ century already showed that averages of n scores correlate higher with each other than the individual scores according to the following equation:

{r_{av}} = \frac{{n*{r_{indiv}}}}{{1 + \left({n - 1} \right)*{r_{indiv}}}}

There are several ways to calculate Shrout and Fleiss’s intraclass correlations, but the most versatile is making use of a mixed effects model, because this easily copes with missing observations (Stevens & Brysbaert, 2016). All we have to do is turn Table 4 in so-called long notation and use a published algorithm. Table 5 shows the first lines of the long notation of Table 4.

**Table 5:** First lines of the long notation of Table 4. Lines with missing values are simply left out.

Participant	Condition	Response

P1	S1	7
P1	S2	5
P1	S3	10
P1	S4	6
P2	S1	9

The R package *psychometric* by Fletcher (2015) contains commands to calculate the two intraclass correlations of Shrout and Fleiss (1979). All you have to do is to import the full Table 5 (long notation) in R and use the following commands:

- library(psychometric)
- ICC1.lme(Response, Participant, data=Table5)
- ICC2.lme(Response, Participant, data=Table5)

This will tell you that the average correlation between repetitions is ICC1 = .39, and that the correlation to be expected between the mean values and the means of another set of four replications is ICC2 = .72. You can see that the value of ICC2 is the one predicted on the basis of the Spearman-Brown equation:

IC{C_2} \approx \frac{{4*IC{C_1}}}{{1 + 3*IC{C_1}}} = \frac{{4*.39}}{{1 + 3*.39}} = .72

This means that we can also use the Spearman Brown equation to estimate how many more stimuli we must present to get a higher reliability for the dependent variable. For this we can use the equation:

n = \frac{{{r_{desired}}*\left({1 - {r_{observed}}} \right)}}{{{r_{observed}}*\left({1 - {r_{desired}}} \right)}}.

In test theory, a value of r = .80 is advised as a good combination of investment and return. This is the value of ICC2 we should aim for and reporting it should be part of every data analysis, also in experimental psychology. To get the value for the data in Table 5, we can calculate how many observations we should add as follows:

n = \frac{{{r_{desired}}*\left({1 - {r_{observed}}} \right)}}{{{r_{observed}}*\left({1 - {r_{desired}}} \right)}} = \frac{{.8*\left({1 - .72} \right)}}{{.72*\left({1 - .8} \right)}} = 1.56.

So, we’d need 1.56 * 4 = 6 observations per participant (or 7 if you want to play safe).

**Figure 5:**
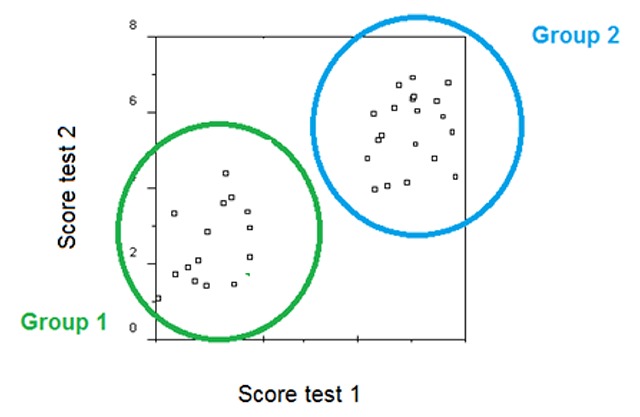
Illustration of how you can find a high correlation in the entire dataset (because of the group difference) and a low correlation within each group (because of the range restriction within groups).

A last issue we must address is how to deal with the fact that experiments consist of several conditions. There are two options: (1) we calculate the ICCs for each condition in the design, or (2) we calculate the ICCs across the complete dataset. In general, both calculations are likely to agree pretty well. However, there will be exceptions. One is illustrated by the valence rating and false memory studies from Table 3, which had a negative correlation between conditions. Obviously, this will hurt the ICC if we calculate it across the entire dataset. Another exception is when there is a big difference between-groups and at the same time not much systematic variance within the groups, as shown in Figure 5. In such a situation you will find strong ICCs across the entire dataset (because of the large group difference) together with weak ICCs in the conditions (because of the restricted range). Finally, design-wide calculations will be worse than condition-specific calculations when there is an interaction between the independent variables, in particular when there is a cross-over interaction.

Because both calculations give us interesting information and are easy to run with contemporary software, it is advised to run (and report) them both in order to get a better feeling for the dataset.

To better appreciate the reliabilities typically observed in psychology, we calculated them for the replication studies reported in Camerer et al. (2018) and Zwaan et al. (2018). For the Camerer et al. studies, we distinguish the repeated-measures designs discussed in Table 3 from the between-groups designs. The results are given in Table 5 (repeated-measures designs) and Table 6 (between-groups designs). The tables also include the reported effect sizes and the effect sizes when the analysis was run on a single observation in each condition. For the repeated-measures experiments these were randomly chosen stimuli per condition; for the between-groups experiments it was the average based on the stimuli used. The two effect sizes help us to understand the increase in effect size by averaging over multiple observations per condition.

**Table 5:** Intraclass correlations for designs with one repeated-measures factor (2 levels). The experiments of Zwaan et al. included more variables, but these did not affect the results much, so that the design could be reduced to a one-way design. The table shows the intraclass correlations, which mostly reach the desired level of ICC2 = .80 when it is calculated within conditions. The table also shows that the average reported effect size was d_z_ = .76. If the experiments had been based on a single observation per condition, the average effect size would have been d_z_ = .24, illustrating the gain that can be made by having multiple observations per participant per condition.

Study	Dependent variable	N parts	N conditions	Nobs_per_ cond	Across the entire dataset		Average within condition		d_N = 1_	d_N = tot_

					ICC1	ICC2	ICC1	ICC2		

*Camerer et al. (2018)*										
Aviezer et al. (2012)	Valence ratings	14	2	88	0.01	0.60	0.23	0.96	0.94	1.43
Kovacs et al. (2010)	Reaction time to visual stimuli	95	2	5	0.41	0.87	0.40	0.76	0.29	0.72
Sparrow et al. (2011)	Reaction time to visual stimuli	234	2	8 & 16	0.10	0.91	0.10	0.81	0.03	0.10
*Zwaan et al. (2018)*										
associative priming	Reaction time to visual stimuli (Session 1)	160	2 × 2 × 2	30	0.28	0.96	0.31	0.93	0.17	0.81
	Reaction time to visual stimuli (Session 2)				0.30	0.96	0.31	0.92	0.18	0.94
false memories	Correct related-unrelated lures (Session 1)	160	2 × 2 × 2	9	0.02	0.29	0.27	0.77	0.20	0.97
	Correct related-unrelated lures (Session 2)				0.06	0.54	0.26	0.75	0.31	1.17
flanker task	RT stimulus congruent incongruent (Session 1)	160	2 × 2 × 2	32	0.40	0.98	0.41	0.96	0.15	0.70
	RT stimulus congruent incongruent (Session 2)				0.29	0.96	0.29	0.92	0.13	0.52
shape simulation	RT to shape matching sentence (Seesion 1)	160	2 × 2 × 2	15	0.42	0.93	0.42	0.91	0.08	0.27
	RT to shape matching sentence (Seesion 2)				0.49	0.97	0.50	0.94	0.17	0.50
spacing effect	Memory of massed v. spaced items (Session 1)	160	2 × 2 × 2	40	0.03	0.54	0.03	0.40	0.20	0.87
	Memory of massed v. spaced items (Session 1)				0.06	0.84	0.07	0.75	0.21	0.92

**Table 6:** Intraclass correlations for between-groups designs. The table shows that the intraclass correlations mostly reached the desired level of ICC2 = .80 when multiple observations were made per condition and all the items used. In general, this improved the interpretation (see in particular the study of Pyc & Rawson, 2010). At the same time, for some dependent variable (e.g., rating scales) rather stable data can be obtained with a few questions (see the study by Wilson et al., 2014).

Study	Dependent variable	N parts	N conditions	Nobs_per_ cond	Across the entire dataset		Average within condition		d_N = 1_	d_N = tot_

					ICC1	ICC2	ICC1	ICC2		

*Camerer et al. (2018)*										
Ackerman et al. (2010)	Evaluating job candicates	599	2	8	0.47	0.87	0.47	0.88	0.10	0.13
Gervais & Norenzayan (2012)	Belief in God	531	2	1	NA	NA	NA	NA	–0.07	–0.07
Karpicke & Blunt (2011)	Text memory	49	2	1	NA	NA	NA	NA	0.83	0.83
Kidd & Castano (2013)	Emotion recognition	714	2	36	0.09	0.78	0.09	0.78	–0.03	–0.08
Morewedge et al. (2010)	M&Ms eaten	89	2	1	NA	NA	NA	NA	0.75	0.75
Pyc & Rawson (2010)	Word translations	306	2	48	0.14	0.89	0.14	0.88	0.12	0.30
Shah et al (2012)	Dots-mixed task	619	2	1	NA	NA	NA	NA	–0.03	–0.03
Wilson et al (2014)	Enjoyment ratings	39	2	3	0.82	0.93	0.73	0.89	1.32	1.44

As predicted, in particular for the designs with repeated-measures, increasing the number of observations per condition made a big difference (Table 5). The estimated effect size if the studies had been based on a single observation per condition was d = .24. Because the number of observations was considerably higher (going from 9 to 88), the obtained effect size was d = .76 and the reliability of the dependent variable was on average .8 (against .2 if based on single observations).

The situation is less compelling for the between-groups designs reported in Camerer et al. (2018), partly because several of the effects could not be replicated and partly because half of the datasets included a single dependent variable, even if it was based on a longer test (including several questions). However, in Table 6 too we see that reliable, stable dependent variables improve the interpretation. The most convincing instance is the replication of Pyc and Rawson (2010). These authors examined the impact of testing on Swahili-English word translation retention. The study was based on 48 translation pairs and the testing effect resulted in a significant effect size of d = .30. If we look at how strong the effect would have been if it was based on singe word pairs, the effect size reduces to d = .12.

Table 6 also illustrates that some dependent variables can be quite reliable even when based on a few items. Wilson et al. (2014) used three items to estimate the enjoyability of a situation (“How enjoyable was the situation?”, “How entertaining was the situation?”, and “How boring was the situation?” – reverse scoring). Intercorrelations between the three ratings were .82, suggesting little improvement for longer questionnaires. At the same time, notice that the reliability can only be assessed when there is more than one measurement and that even in this study the average of the three questions still increased the reliability to .93.

Given that a correlation of r = .8 multiplies d_z_ by 1.5, for repeated-measures studies with reliable dependent variables we can postulate d_z_ = .6 rather than d_z_ = .4. Tables 7 and 8 show the impact this has on the numbers of participants needed for studies with 80% power. Remember, however, that postulating an effect size of d_z_ = .6 requires reassurance that the dependent variable has a reliability of at least .8. This can easily be verified (and reported) by using ICC2.

**Table 7:** Numbers of participants required for various designs when d = .4, .5, and .6 and the data are analyzed with traditional, frequentist statistics (p < .05). The numbers of the d = .4 column are the default numbers to use. The higher values of d require dependent variables with a reliability of .8 at least. Therefore, authors using these estimates must present evidence about the reliability of their variables. This can easily be done by calculating the ICC1 and ICC2 values discussed above.

Traditional, frequentist analysis (p < .05)			

	d = .4	d = .5	d = .6

1 variable between-groups			
• 2 levels	**200**	130	90
• 2 levels, null hypothesis	**860**	860	860
• 3 levels (I = II > III)	**435**	285	195
• 3 levels (I > II > III)	**1740**	1125	795
1 variable within-groups			
• 2 levels	**52**	34	24
• 2 levels, null hypothesis	**215**	215	215
• 3 levels (I = II > III)	**75**	50	35
• 3 levels (I > II > III)	**300**	195	130
Correlation	**195**	125	85
2 × 2 repeated measures			
• Main effect one variable	**27**	18	13
• Interaction (d v. 0)	**110**	75	50
2 × 2 split-plot			
• Main effect between			
◦ r = .5	**150**	100	70
◦ r = .9	**190**	120	90
• Main effect repeated-measure	**55**	34	24
• Interaction (d v. 0)	**200**	130	90
• ANCOVA			
◦ r_rep_measure_ = .5	**160**	100	70
◦ r_rep_measure_ = .9	**200**	130	90

**Table 8:** Numbers of participants required for various designs when d = .4, .5, and .6 and the data are analyzed with Bayesian statistics (BF > 10). The numbers of the d = .4 column are the default numbers to use. The higher values of d require dependent variables with a reliability of .8 at least. Therefore, authors using these estimates must present evidence about the reliability of their variables. This can easily be done by calculating the ICC1 and ICC2 values discussed above.

Bayesian analysis (BF > 10)			

	d = .4	d = .5	d = .6

1 variable between-groups			
• 2 levels	**380**	240	170
• 2 levels, null hypothesis	**2400**	2400	2400
• 3 levels (I = II > III)	**690**	450	300
• 3 levels (I > II > III)	**2850**	1800	1200
1 variable within-groups			
• 2 levels	**100**	65	45
• 2 levels, null hypothesis	**720**	720	720
• 3 levels (I = II > III)	**125**	80	55
• 3 levels (I > II > III)	**540**	340	240
Correlation	**370**	230	160
2 × 2 repeated measures			
• Main effect one variable	**52**	32	23
• Interaction (d v. 0)	**210**	130	85
2 × 2 split-plot			
• Main effect between			
◦ r = .5	**290**	190	130
◦ r = .9	**360**	220	160
• Main effect repeated-measure	**100**	66	46
• Interaction (d v. 0)	**390**	250	170
• ANCOVA			
◦ r_rep_measure_ = .5	**300**	190	130
◦ r_rep_measure_ = .9	**380**	230	170

## Increasing the power to 90%

So far, we have discussed the numbers required for 80% power. That is, we have 80% chance of finding statistical significance in the sample test if the effect is present at the population level. In 20% of the cases we fail to find the effect. In other words, failing to find the effect (20%) is considered less important than obtaining a fluke effect that does not exist in reality (5% when p < .05).

However, we can think of situations in which failing to find a true effect has important costs as well. Think of a scientific theory that critically depends on a particular effect. Are we then relaxed at having an a priori chance of 20% of not finding the effect even though it exists in reality? Or would we feel better if the prior chance was 10% or even 5%? How costly is it to give up a theory because we failed to put in enough effort to test it properly?

So, there may be good reasons to go for a power of 90% or even more. Unfortunately, this comes at a further cost in terms of the number of participants that must be tested. Table 9 gives the numbers needed to detect d = .4 with power = .9 and p < .05 or BF>10 (two-tailed tests). These numbers can be compared to those of Tables 7 and 8.

**Table 9:** Numbers of participants required for various designs when d = .4 and power is increased to 90%. The latter decreases the chances of not finding an effect present in the population.

	d = .4, power = .9, p < .05	d = .4, power = .9, BF > 10

1 variable between-groups		
• 2 levels	264	480
• 2 levels, null hypothesis	1084	3600
• 3 levels (I = II > III)	570	840
• 3 levels (I > II > III)	2160	3450
1 variable within-groups		
• 2 levels	70	130
• 2 levels, null hypothesis	271	1800
• 3 levels (I = II > III)	100	150
• 3 levels (I > II > III)	360	610
Correlation	260	460
2 × 2 repeated measures		
• Main effect one variable	35	65
• Interaction (d v. 0)	145	270
2 × 2 split-plot		
• Main effect between		
◦ r = .5	200	360
◦ r = .9	250	450
• Main effect repeated-measure	70	130
• Interaction (d v. 0)	300	540
• ANCOVA		
◦ r_rep_measure_ = .5	210	360
◦ r_rep_measure_ = .9	260	460

## Discussion and recommendations

Articles (and talks) about power in psychology research have a predictable course. At first the audience is engaged and enthusiastic about the need for properly powered studies. This suddenly changes, however, when the required numbers of participants are tabulated. Then howls of disbelief and protest arise. Surely the numbers must be wrong! Effect sizes in psychology must be larger than the assumed d = .4! For some classic, robust effects in designs with repeated-measures and many observations per level this is indeed true (e.g., Zwaan et al., 2018). However, time and time again, an effect size of d = .4 (r = .2) comes out as the average effect size in psychology (Gignac, & Szodorai, 2016; Open Science Collaboration, 2015; Stanley et al., 2018), meaning that half of the effect sizes are likely to be even smaller. So, unless one has good evidence to the contrary, d = .4 should be our guide in setting up new research. This leads to following recommendations.

### For many research questions, studies with less than 100 participants are underpowered

Tables 7, 8, 9 summarize the numbers needed for various popular designs in psychology, if we assume an effect size of d = .4. As can be seen, very little research can be done properly with samples lower than N = 100 participants per between-subjects group. The only exception is the main effect of a repeated-measures variable with two levels analyzed with frequentist statistics (p < .05). This can be investigated sufficiently with a sample size of N ≈ 55. For other analyses, we require N ≈ 100 (repeated-measures variable with three levels, interactions of repeated-measures variables, a two-level within-subjects variable analyzed with the default versions of Bayesian software packages), N ≈ 200 (a between-groups variable with two levels, an interaction between a repeated-measures variable and a between-groups variable), or even larger Ns (in particular for the Bayesian analyses and when we want to increase the power to 90%).

The numbers required for 80% powered studies are considerably higher than current practice, which is the reason why we keep on having underpowered studies in the literature (Stanley et al., 2018; Szucs & Ioannides, 2017a). Still, they are not impossible, as shown in recent replication projects, which often exceed the minimum sample sizes. One reason why larger sample sizes are easier to run nowadays than before is that studies are increasingly administered via the internet (Birnbaum, 2004; Gosling & Mason, 2015; Gosling, Vazire, Srivastava, & John, 2004; Hauser & Schwarz, 2016; Hilbig, 2016; Litman, Robinson, & Abberbock, 2017; Paolacci & Chandler, 2014). It would be a lost opportunity if this new technique were used to run an even larger number of underpowered studies (like happened with the introduction of the personal computer for stimulus presentation and data analysis; Brysbaert & Stevens, 2018) rather than more well-powered studies.

Particularly worrying for cognitive psychology is the large number of observations needed to properly test the interaction between a repeated-measures variable and a between-groups variable in the split-plot design. It looks like this effect needs the same number of participants as a between-groups comparison, something which has not been documented before. An analysis of replication studies suggests that in particular between-subjects manipulations are difficult to replicate (Schimmack, 2015), raising the possibility that the same may be true for interactions between repeated-measures and between-groups variables.

### Stopping the curse of underpowered studies requires a different reward system

The main reason why underpowered studies keep on being published is that the current reward system favors such studies. As long as editors, reviewers, supervisors, and examiners endorse studies with statistically significant effects independent of power, researchers chasing sensational new findings with small samples will win the publish or perish race in academia (Edwards & Roy, 2017; Fraley & Vazire, 2014; Higginson, & Munafò, 2016; Munafò, et al., 2017; Smaldino & McElreath, 2016).

One element that could help nudging researchers towards properly powered studies may be the inclusion of power in the badge system used in some journals to promote good research practices (Chambers, 2013; Lindsay, 2015, 2017; Kidwell et al., 2016; Nosek & Lakens, 2014). Only studies meeting the standards for an 80% or better powered study would qualify.

Also the evaluation of graduate students will have to change. Rather than being impressed by a series of 10-20 small-scale studies, supervisors and examiners should start endorsing PhD theses with 2–4 properly run studies.

Finally, well-powered studies are less insurmountable when they are done in collaboration with other research groups. It is probably not a co-incidence that many replication studies are run in labs with active collaboration policies. This is another element that can be taken into account when evaluating research.

### About the different numbers for p < .05 and BF > 10

A comparison of Tables 7, 8, 9 is likely to bias readers against Bayesian analysis, given their fondness for the smallest possible sample sizes. This is not justified. Promotors of Bayesian analysis have opted for a more stringent criterion to accept ‘significant’ findings, because this reduces the chances of non-replicable false positives being published. Hence the larger numbers of participants required.

If the criterion for significance in Bayesian analysis is placed at BF > 3 (moderate evidence in favor of the hypothesis), then the numbers of participants required for properly powered studies are close to those for p < .05. Indeed, some authors have suggested that the Bayesian approach may be more acceptable if the criterion were set at BF > 3 (Dienes, 2016) or at BF > 6 as an in-between compromise (Schönbrodt, Wagenmakers, Zehetleitner, & Perugini, 2017).^14^ In contrast, other authors have argued that the psychological literature would be more trustworthy if the criterion for significance in traditional tests were set at p < .005 instead of p < .05 (Benjamin et al., 2018). This requires sample sizes closer to those of Table 8 than to those of Table 7. For instance, the numbers of participants required for a between-subjects t test at p < .005, two-sided, is N = 169 per group (or N = 338 in total). For a within-subjects t test the number is N = 88.

High criteria (p < .005, BF > 10) are particularly needed when researchers are investigating a new, theoretically surprising or counterintuitive hypothesis. If a lenient criterion is used for such studies (p < .05) it can be shown that statistically ‘significant’ findings are more likely to be false negatives than genuine findings, certainly when they are based on underpowered studies (Colquhoun, 2014). We also saw that null hypothesis testing in Bayesian analysis requires high numbers of participants even for BF < 1/3. On the other hand, p < .05 and BF > 3 may be more appropriate for a series of converging studies based on solid theory, as otherwise the number of participants to be tested may become needlessly high (Loiselle & Ramchandra, 2015). As a result, some researchers have recommended to use the criterion flexibly and to justify the alpha level used (Bishop, 2018; Lakens et al., 2018).

At this point, it is important to mention that proponents of the Bayesian approach are not overly enthusiastic about the yes/no null hypothesis significance testing discussed in the present article, for good reasons (e.g., Cumming, 2014; Gigerenzer & Marewski, 2015; Kruschke & Liddell, 2018). The Bayesian approach is oriented more towards estimation of model parameters and reducing the accompanying uncertainty by continuous fine tuning than towards binary null hypothesis testing based on non-informative priors. As such, the present article gives a skewed picture of Bayesian analysis because of its emphasis on the power of statistical tests (see Kurschke & Liddell, 2018, for a wider picture; also see Szucs & Ioannidis, 2017b, for the limits of null hypothesis significance testing within the traditional frequentist approach). Still, it is important that users of software packages have knowledge of the power requirements when they use Bayesian factors to argue for a null hypothesis or an alternative hypothesis. Observing a large or small BF is as uninformative as a small p-value, when based on a hopelessly underpowered study (see the introduction). Indeed, in all our simulations we observed some very high and very low BFs for d = .4 both for underpowered and properly powered studies. So, the BF-value obtained in a specific study (simulation) is uninformative about the methodological soundness of the study. Power must be based on an analysis made before the experiment is run, not after the results are obtained (Hoenig & Heisey, 2001).

### The numbers of Tables 7 and 8 are minimum numbers

It is also good to stress once again that the numbers in Tables 7 and 8 are not maximum numbers (an ideal to strive for), but minimum numbers for 80% powered studies under the constraints given. Having more participants is good, having less is bad. There are four main reasons.

First, the data in Tables 7 and 8 have been obtained under ideal simulations. In everyday life the data are likely to be messier and violate some requirement of the statistical test (e.g., normal distribution, balanced designs, independence of observations, no extraneous fluctuating sources of noise, etc.). When based on enough data, small violations are unlikely to invalidate the conclusions much (unless a strong confound has been overlooked). However, they will introduce extra noise and so require a few more observations to reach the same level of power as under ideal circumstances.

Second, an average effect size of d = .4 means that half of the expected effect sizes are smaller. So, a prudent researcher may rightly wish to examine a new phenomenon under the assumption of d = .3 or even d = .2. Such practice can easily be defended, but it comes at a cost. For d = .3, the numbers of the d = .4 columns in Tables 7 and 8 must be multiplied by 1.75. For d = .2 they must be multiplied by almost 4. It is unlikely that such numbers will be rewarded within the current academic system (although this hopefully will change once power issues are given due consideration). Furthermore, it is good to keep in mind that effect sizes of d < .4 have little practical impact. So, one needs good theoretical motivation to go after such effects, in which case one is likely to know the direction of the effect, so that one-tailed tests can be used (these require fewer participants).^15^

Third, Tables 7 and 8 strive for a power of .8. This means that as an experimenter you accept 20% chance of not finding the effect you are after, although it exists. As Table 9 shows, this need not be the case. Nothing prevents you from going for a higher power (.9 for instance), except that it requires extra participants.

Fourth, we can look at how precisely the numbers of Tables 7 and 8 measure the effects. To some extent, the issue of statistical significance is of secondary importance in science. Research is more than a series of yes/no answers based on hypothesis testing. The main aim of research is to establish the *magnitude* of the effects and how exactly they interact with each other (Kelley, Maxwell, & Rausch, 2003; Trafimow & Myüz, in press). When we look at how precisely the effect sizes are estimated in Tables 7 and 8, the outcome is rather sobering. For the between-groups t test in Table 7 (d = .4 with 100 participants per condition) we get a confidence interval of plus or minus .3. For the repeated-measures design with 52 participants, the confidence interval is even larger: plus or minus .4. This means that in terms of precision the experiment says little more than that the effect can vary from nearly non-existent (d slightly above .0) to very big (d = .8). Bayesian statistics has a credible interval indicating the likely range of values within which the estimated parameter falls (see Morey, Hoekstra, Rouder, Lee, & Wagenmakers, 2016, for a discussion of the credible interval vs. the confidence interval). With the numbers for d = .4 in Table 8, we see that the credible interval for an effect in a between-groups t test ranges from minus .2 to plus .2. For a repeated-measures t test it ranges from the effect minus .3 to the effect plus .3. These numbers are better than the confidence intervals of Table 7 (because of the larger numbers of participants), but still tell us that in terms of precision the participant numbers in Tables 7 and 8 are at the low end rather than at the high end. Trafimow and Myüz (in press) argue that we need at least 385 participants per condition if we want to measure the mean with a precision of .1 standard deviation.

Psychology really could do with more high-powered, high-precision studies of important phenomena, like the top studies of Figure 2. This is one of the aims of current replication attempts (LeBel, McCarthy, Earp, Elson, & Vanpaemel, in press), but it should be the ambition of all good researchers.

At the same time, it is important to comprehend that the numbers of Tables 7, 8, 9 must not be used as fixed standards against which to judge each and every study without further thought. A better analogy is that of reference numbers. If you have reasons to believe the smallest effect size of interest is d = .4 and if you want to use the analyses included in the tables, then you must go for the numbers in the tables. If, however, you have reasons to believe that the effect size under investigation is smaller but of theoretical interest, you will require higher numbers. Alternatively, if you have good evidence that the expected effect size is larger, you can justify smaller numbers. So, it is not of primary importance that you slavishly follow/impose the numbers given in the tables, but that you justify adequately why the number tested/requested deviates from that in the table. Importantly, such justification must be done before the study is run, not after seeing the data and deciding whether they agree with your expectations.

### Increasing the power of an experiment by having multiple observations per participant per condition

An aspect often overlooked in power analyses is that noise can be reduced by having more observations per participant. This is particularly effective for repeated-measures designs, because the power of such designs depends on the correlation between the conditions in addition to the difference in means (i.e., the distinction between d_z_ and d_av_). When the correlation is r = .8, d_z_ ≈ 1.5 * d_av_; when r = .9, d_z_ ≈ 2 * d_av_. This brings research in the realm of the d = .5 and d = .6 columns in Tables 7 and 8, where smaller sample sizes are acceptable.

Brysbaert and Stevens (2018) recommended 40 participants and 40 stimuli per condition as good practice in reaction time studies with two repeated-measures conditions. Looking at Table 7, we see that this assumes an effect size between d = .4 and d = .5. For an effect size of d = .4, a recommendation of 52 participants would have been better. Unfortunately, Brysbaert and Stevens (2018) further suggested that the numbers of 40 participants and 40 stimuli per condition may be true for more complex designs. A look at Table 7 makes this unlikely. Even simple interactions already require 100 participants (when the interaction involves two repeated-measures variables) or even close to 200 participants (when the interaction includes a between-groups variable), unless the effect sizes can be shown to be in the realm of d_z_ = .5 and d_z_ = .6. Further simulations will have to indicate whether these designs also require more stimuli per condition.

The correlation between two within-participant conditions can be calculated and depends on the reliability of the measures. Therefore, it is good practice to measure and optimize reliability. Reliability can be measured with the intraclass correlation; it can be optimized by increasing the number of observations. The latter is particularly required for noisy dependent variables, such as reaction times. Dependent variables with less variability (e.g., Likert ratings) may require fewer observations. Reliability can only be calculated when there are at least two observations per participant per condition. Researchers are recommended to make sure this is the case either by repeating the stimuli or by including equivalent items.

It would be good practice if researchers always included the effect sizes d_z_ and d_av_ when they report a pairwise test of repeated-measures conditions. This allows readers to calculate the correlation between the conditions and gives makers of meta-analyses all the information they need to compare between-groups studies with repeated-measures studies. Table 10 shows the values for the paradigms investigated by Zwaan et al. (2018). A comparison of d_z_ with d_av_ allows readers to deduce the correlations between the conditions (Table 3). When d_z_ > d_av_, the correlation is higher than r = .5; otherwise it is less.

**Table 10:** Comparison of d_z_ and d_av_ for repeated-measures designs in Zwaan et al. (2018). They show that d_av_ < d_z_ when r = .5 (see Table 3). Reporting d_av_ in addition to d_z_ for pairwise comparisons allows readers to compare the effect sizes of repeated-measures studies with those of between-groups studies.

Study	Dependent variable	N parts	N conditions	Nobs_ per_cond	dz_N=1_	dz_N = tot_	d_av_

*Zwaan et al. (2018)*							
associative priming	Reaction time to visual stimuli (Session 1)	160	2 × 2 × 2	30	0.17	0.81	0.37
	Reaction time to visual stimuli (Session 2)				0.18	0.94	0.35
false memories	Correct related-unrelated lures (Session 1)	160	2 × 2 × 2	9	0.20	0.97	1.66
	Correct related-unrelated lures (Session 2)				0.31	1.17	1.76
flanker task	RT stimulus congruent incongruent (Session 1)	160	2 × 2 × 2	32	0.15	0.70	0.44
	RT stimulus congruent incongruent (Session 2)				0.13	0.52	0.20
shape simulation	RT to shape matching sentence (Seesion 1)	160	2 × 2 × 2	15	0.08	0.27	0.13
	RT to shape matching sentence (Seesion 2)				0.17	0.50	0.21
spacing effect	Memory of massed v. spaced items (Session 1)	160	2 × 2 × 2	40	0.20	0.87	1.00
	Memory of massed v. spaced items (Session 2)				0.21	0.92	0.87

### Taking power more seriously as reviewer, editor, and textbook writer

The curse of underpowered studies is unlikely to stop as long as reviewers and editors value potential interest of the findings more than methodological soundness. The main problem with the evaluation of findings after the experiment is run is that most significant findings can be given an interesting post hoc interpretation. Effects in line with the expectations pass without question and are interpreted as adding credibility to the methodological choices made in the study (in line with the post hoc ergo propter hoc reasoning fallacy). Significant interactions ‘suggest’ that the effect is true for one group of participants only or for one type of stimuli only (whereas such interactions can easily be false positives). Even unexpected effects may point to exciting new insights. Non-significant findings cause more interpretation problems and, therefore, are less likely to get published, leading to the file drawer problem and a failure to expose previously published false positives.

Logistically, it is extremely simple to include power considerations into the editorial decision. If editors and reviewers collectively decided no longer to publish underpowered studies, research practices would change overnight. That this has not happened yet, is arguably due to two factors: (1) the underestimation of the power issue, and (2) the lack of clear guidelines about the sample sizes needed for properly powered studies. The present article is an attempt to address the second factor. It is hoped that it will kick off the discussion and lead to a consensus paper with a wider remit than a single-authored publication.

The proposal is that the numbers of the d = .4 columns in Tables 7 and 8 are the default values to judge whether a study meets the power requirements for frequentist or Bayesian null hypothesis significance testing. The d = .5 and d = .6 columns can be used if the authors give good reasons why their effect sizes are larger than average. These reasons must not make reference to existing research traditions or data from previous small-scale studies, but can be data from large-scale studies or data indicating that the reliability of the measures is high enough to have d_z_ sufficiently larger than d_av_. It is suggested that studies with numbers of participants lower than those required for d = .6 are not considered for publication in good journals.

The numbers in Tables 7, 8, 9 also provide textbook writers with useful guidelines about whether to include a study in their book and how best to describe the finding: As a well-established fact or an interesting hypothesis that still requires a proper test? Indeed, the replication crisis has shown that textbook writers must be much more hesitant calling a finding “scientific” as long as it is based on underpowered studies.

### What about research topics in small populations or with expensive testing?

The numbers in Tables 7, 8, 9 seem to exclude research on all topics involving small populations or research techniques with a hefty cost. Does this mean that psychology can only investigate issues lending themselves to large-scale, cheap internet testing?

To answer this question, we must return to the consequences of underpowered studies. Most of the time, these studies will not detect a true effect (Table 1). If they detect it, this is because the effect size in the small sample happens to be considerably larger than the true effect size. There is also a non-negligible chance if a “significant” effect is found that it does not exist at the population level. Chances of non-replicable, spurious effects increase the more complex the design is, certainly when no correction for multiple testing is made (Maxwell, 2004). The situation is further complicated by the presence of “nearly significant” effects, tempting researchers to offer even more “explanations” for “potentially interesting” patterns that do not exist in reality.

As a result, underpowered studies are unlikely to add much insight. They may hit on a new true finding if the sampling error happens to enlarge the effect in the small sample studied, but most of the time they will just churn out negative findings and false positives.

So, rather than continuing to excuse underpowered studies, we must use the information in Tables 7, 8, 9 to determine what type of research is meaningful with small samples. The tables are very clear in this respect: Only a main effect between two reliably measured within-subjects conditions is within reach. The more uncorrelated situations over which the main effect is observed, the fewer participants are needed (see the smaller number of participants required for a 2 × 2 repeated measures design than for a t-test with related samples). Further reassuring is that the main effect is not compromised by the presence of control variables (e.g., the order in which the conditions are presented or the stimulus list used), as long as significant effects due to these control variables are not interpreted and at a minimum have p-values corrected for multiple testing.

In sum, researchers confronted with a small number of participants must not search for excuses to keep doing bad research, but must ask questions that stay informative (i.e., related to a main effect between two within-subjects conditions). For them, even more than for others, it is imperative to try to measure each participant as thoroughly as possible, so that stable results are obtained per participant. It may even be questioned whether group tests such as t-tests and ANOVAs are the best designs. It may be better to use a combination of single-case experiments, to get the best possible estimates of effects at the level of the individual, and meta-analysis to extend the conclusions to a larger population (Onghena, Michiels, Jamshidi, Moeyaert, & Van den Noortgate, 2018; Shadish, Hedges, & Pustejovsky, 2014; Smith, 2012).^16^ Ironically, this is very much a return to the first studies in experimental psychology, as these often were intensive studies on small samples (e.g., in psychophysics). A challenge for these studies is to make sure that they are not invalidated by demand characteristics.

Alternatively, we must start budgeting for appropriate numbers of participants. If little good research can be done with samples smaller than 100 participants, we must mention this in grant applications and argue why the money is needed. Better to fund useful research than to pay for studies that burden the field with more ambiguity after they are run than before.

### Going beyond Tables 7, 8, 9

A frustration with tables is that they often do not cover the exact condition you are interested in. This is the attraction of power calculators giving you explicit numbers for whatever combination of independent variables and power requirements you can think of, even though you may not understand the outcome in the way the authors assumed you would do. In this respect, it may be interesting to know that the numbers in Tables 7, 8, 9 are not so difficult to obtain. Basically, what you need is:

– An algorithm generating random numbers that are normally-distributed and (for repeated-measures) correlated,
– An algorithm that runs the statistical test you are interested in,
– A loop to do this a few hundred/thousand times and summarize the findings.

All of this is rather straightforward in computer languages with statistical libraries, such as R. To help you on your way, all programs used for the simulations in Tables 7, 8, 9 are available at https://osf.io/8uaxb/. They can easily be adapted for different parameters. With a bit of tinkering, you can also adapt them for other designs (e.g., a 2 × 3 repeated-measurements design).

What you have to do, is make your predictions explicit in terms of standardized values for the various conditions and the ways in which you are going to analyze your data (exactly the things you do when you preregister a study). By then generating and analyzing multiple datasets following these restrictions, you can estimate how often the statistical analysis will confirm the structure you used to generate the numbers (and which you think represents reality). In that way, you immediately get a feeling for the usefulness of your design. It may even be an idea to drop the automatic loop and generate, analyze, and report the data of 100 simulations one after the other, just to get an idea of how often you obtain results that do not agree with the structure you imposed on the number generator. Finding and reporting 90 simulations that fail to confirm a structure you know to be there arguably is the best remedy to avoid underpowered studies.

## Footnotes

Baayen’s original text was about p-values, which suffer from the same problems.

As everything we do is fallible, some of these numbers may turn out to be too optimistic or pessimistic. The hope is that by making them explicit the research community can zoom in on them and fine tune them if needed.

As can be seen in the many articles starting out with a reasonable sample size, but ending up with an underpowered study by the time all problematic participants and observations have been dropped.

As I experienced myself. For instance, Callens, Tops, and Brysbaert (2012) was rejected by two American journals, because the reviewers felt unsure about the testing language (Dutch). This concern outweighed the size of the study (which involved two years of solid data collection and analysis).

A limitation of this study was that it asked about between-groups designs, whereas most cognitive psychologists are more familiar with repeated-measures designs.

The numbers coming out of the simulations were already so grim, that the author did not have the heart to further impose a criterion of 90% power as standard practice.

As Borsboom (2013) noticed, having a theoretical understanding of the underlying principles is the ideal case because a good theory allows you to infer what would happen to things in certain situations without having to create the situations. For instance, you can figure out whether an imaginary bridge will stand or collapse in imaginary conditions. Without a theory, if you want to know what would happen if you did X, you actually have to do X, which is more labor-intensive and less insightful. Luckily, computers nowadays are so powerful and fast that we can easily run the simulations needed for the power analyses in the present article. An advantage of simulations is that they are less prone to oversight of important details (e.g., it is becoming clear that for a long time we misunderstood the power of interactions, as will be discussed later). So, the present paper is a combination of findings based on theoretical analysis (for the simple cases) and simulation (for the more complex cases).

One-tailed t-test p < .05 (so, .1 two-tailed), Bonferroni correction for three pairwise comparisons.

In which case we would have found that f = .1886 for a design with .0, .0, and .4; and f = .1633 for a design with .0, .2, and .4.

This may be another reason why some researchers have unrealistic expectations about the power of experiments.

The reason why the equation is only approximate is that d is calculated on N and η^2^_p_ on df. If we calculate d on df, we get d = {\textstyle{t \over {\sqrt {df}}}} = {\textstyle{{3.04} \over {\sqrt 9}}} = 1.01. The larger N, the closer the approximation of the equation. It is also possible to make the equation more complex, so that it always gives the correct value.

Vasilev et al. (2018) reported a correlation of r = .74 between reading rate in silence and reading rate in noise in the repeated-measures studies they reviewed.

A more precise way to know the weights of the main effects and the interaction effect, is to use multiple regression analysis with dummy codes for the independent variables (–1 and +1 work best). By multiplying the codes of Factor A and Factor B, you get the interaction. Then you can run a multiple regression with three continuous predictors: Factor A, Factor B, and the interaction. The regression weights will give you sizes of the three effects that can be compared directly. This requires the data to be coded in long notation (see later in this article).

Schönbrodt et al. (2017) contains a sequential hypothesis Bayesian testing approach, which they argue requires fewer participants than listed here without increasing the probability of false positives (a similar argument is made by Lakens, 2014, for frequentist sequential analyses). This alternative approach is not included in the present article because of the dismal record psychologists have when given the opportunity to peek at the data. We really must learn to accept that the only way to get properly powered experiments with good effect size estimates is to test more than a few participants. It is not clear either how well the sequential technique works for the more complicated designs with multiple pairwise comparisons.

For instance, a t-test for related samples with d = .2, p < .005 requires N = 296 participants for a one-tailed test, against N = 337 for a two-tailed test.

These methods are becoming increasingly familiar, as they involve linear mixed effects models, which are on their way to become the standard in experimental psychology.
